# Novel Bioinspired Quercetin-Based Polymers for the Sustained Release of Donepezil in Alzheimer’s Disease Therapy

**DOI:** 10.3390/polym18020234

**Published:** 2026-01-16

**Authors:** Elisabete P. Carreiro, Pedro Múria, Diogo Velez, Manuela R. Carrott, Anthony J. Burke, Ana R. Costa

**Affiliations:** 1 Institute for Research and Advanced Training (IIFA), LAQV-REQUIMTE, University of Évora, Rua Romão Ramalho, 59, 7000-671 Évora, Portugal; manrc@uevora.pt; 2 Department of Chemistry and Biochemistry, School of Science and Technology, University of Évora, 7000-671 Évora, Portugal; pmuria@unilicungo.ac.mz (P.M.); diogofrazao31@gmail.com (D.V.); 3 Coimbra Chemistry Centre, Institute of Molecular Sciences (CQC-IMS), Departamento de Química, University of Coimbra, 3004-535 Coimbra, Portugal; ajburke@ff.uc.pt; 4 Pharmaceutical Chemistry Laboratory, Faculty of Pharmacy, Pólo das Ciências da Saúde, University of Coimbra, Azinhaga de Santa Comba, 3000-548 Coimbra, Portugal; 5 CNC-UC-Center for Neuroscience and Cell Biology, CIBB-Center for Innovative Biomedicine and Biotechnology, University Coimbra, 3004-504 Coimbra, Portugal; 6 Department of Medical & Health Sciences, School of Health & Human Development, University of Évora, 7000-810 Évora, Portugal; acrc@uevora.pt; 7 IIFA, CREATE-Center for Sci-Tech Research in Earth System and Energy, University of Évora, 7000-810 Évora, Portugal

**Keywords:** molecularly imprinted polymers, drug delivery, quercetin derivative, donepezil, fluorescent polymers, pH-responsive materials, Alzheimer’s disease

## Abstract

This work was inspired by quercetin, a natural bioflavonoid with well-known neuroprotective properties. We synthesized a new functional monomer, 3-acryloxy-3′,4′,5,7-tetramethylquercetin **1**, and used it to prepare, for the first time, a molecularly imprinted polymer (MIP) selective for donepezil, the main drug used in Alzheimer’s disease therapy. The polymer was designed to be fluorescent and responsive to pH changes, aiming for controlled drug release. The optimized **MIP-4**, produced from a 1:1 mixture of the monomer **1** and acrylic acid, was characterized by FTIR-ATR, fluorescence spectroscopy, SEM, and DLS, confirming its chemical composition, morphology, particle size distribution and zeta potential. Adsorption studies showed higher donepezil binding capacity for MIP than for NIP, highlighting the polymer’s selective recognition. In vitro release experiments at pH 3, 5.5, and 7 revealed a pH-dependent behaviour, with nearly 98% cumulative donepezil release at pH 7. The polymer was non-cytotoxic and successfully released donepezil in *in vitro* assays, enabling effective inhibition of *ee*AChE. These results provide a proof of concept supporting the potential of quercetin-derived fluorescent molecularly imprinted polymers as selective and stimuli-responsive platforms for donepezil delivery.

## 1. Introduction

Alzheimer’s disease (AD) is currently one of the most concerning diseases worldwide, affecting millions of people and remaining incurable [1]. AD is a progressive and multifactorial neurodegenerative disease, whose pathophysiology involves multiple interrelated mechanisms, including beta–amyloid peptide aggregation and tau protein hyperphosphorylation, with underlying cholinergic dysfunction and oxidative stress, which together lead to neuronal damage and cognitive decline [2,3,4]. Despite all the efforts made by researchers and the significant financial investment in the search for a cure for this complex disease, the main treatments available continue to be essentially a response to symptoms, aiming only to improve neurotransmission and slow the progression of the disease.

Donepezil, a reversible acetylcholinesterase inhibitor, is among the group of therapeutic strategies for Alzheimer’s Disease (AD) and is used as a first-line treatment to control the cognitive symptoms of the disease. However, despite its clinical efficacy, donepezil has several pharmacokinetic limitations, including a relatively short half-life, extensive hepatic metabolism, and low brain bioavailability due to restricted permeability across the blood–brain barrier (BBB). As a result, a large portion of the drug acts peripherally, leading to off-target cholinergic effects that compromise patient adherence [5]. Due to the lack of a cure and the limitation of available treatments, there is an urgent need to develop new delivery systems capable of improving drug stability, controlling and regulating release kinetics, thereby improving therapeutic outcomes while at the same time minimizing side effects. To date, various drug delivery systems (DDS) for donepezil have been developed for oral, transdermal, and intranasal administration; however, all these approaches still have significant limitations, including low bioavailability, variable absorption, and difficulties in maintaining stable plasma concentrations [6]. These limitations highlight the urgent need to develop innovative strategies to address these problems.

Natural bioactive compounds have the potential to act as adjuvants in the therapy of neurodegenerative diseases. Among them is quercetin (QUER) (3,3′,4′,5,7-pentahydroxyflavone), which is a flavonoid naturally present in many fruits and vegetables, and has a wide range of biological activities already described, including antioxidant, anticancer, anti-inflammatory, neuroprotective, and anti-Alzheimer effects [7,8,9,10]. Due to its potential to alleviate neurodegenerative processes, quercetin has been widely explored as a candidate for therapies for Alzheimer’s disease (AD). It is well known that quercetin reduces oxidative stress, prevents mitochondrial dysfunction, inhibits β-amyloid aggregation, and cholinesterase activity. However, its clinical application is limited by low water solubility and low bioavailability, which has prompted the exploration of polymer-based encapsulation and delivery systems, such as liposomes and nanoparticles [11,12,13].

In recent years, the combination of conventional drugs with bioactive natural compounds has emerged as a promising strategy for the treatment of complex diseases such as cancer and Alzheimer’s disease. This approach may allow for better therapeutic outcomes with lower doses, reducing toxicological potential, while enabling simultaneous action on multiple pathways, constituting a possibility for a more effective and safer treatment [14,15,16,17,18].

Molecularly imprinted polymers (MIPs), also known as artificial antibodies, are polymers with a “memory” for a specific template molecule. Their preparation involves the self-assembly of the template with functional monomers in a porogenic solvent, followed by polymerisation in the presence of a crosslinker, leading to the formation of cavities with specific recognition for the template molecule [19,20]. Owing to their privileged physicochemical properties and ease of preparation, these materials have been extensively explored across several key areas, including the food safety analysis [21,22,23,24,25,26], environmental remediation [27,28], sensors [29], biomedical diagnostics, drug delivery and therapeutics [30,31,32,33,34,35,36], among others.

MIPs have emerged as a versatile platform for controlled drug delivery due to their high selectivity, chemical stability, and adjustable properties. By creating template-specific cavities, MIPs enable selective recognition and sustained release of target molecules [31,32,33]. Although some MIP-based delivery systems for donepezil have been reported, they have relied exclusively on synthetic monomers [34,35,36].

In this work, we aim to develop a molecularly imprinted polymer (MIP) based on natural products with selectivity for donepezil, where the natural monomers can enhance the interaction of the drug with its molecular target. Moreover, besides being environmentally friendly and biocompatible by nature, the use of bioactive or natural monomers in MIP synthesis could provide additional pharmacological benefits, intrinsic of those biomolecules, such as antioxidant and anti-inflammatory activity, which are particularly desirable in neurodegenerative disorders characterized by oxidative stress. In this context, we developed molecularly imprinted polymeric particles using a quercetin-derived monomer, 3-acryloxy-3′,4′,5,7-tetramethylquercetin **1**, to achieve selective recognition and sustained release of donepezil. The resulting polymers were evaluated for drug binding capacity, release kinetics, cholinesterase inhibition, cytotoxicity, and antioxidant activity. This approach represents an innovative strategy to integrate drug delivery and intrinsic pharmacological functionality, providing a multifunctional platform for the controlled and biocompatible delivery of donepezil in Alzheimer’s therapy.

## 2. Materials and Methods

### 2.1. General Conditions

The solvents and reagents used in this study were supplied by Sigma-Aldrich (St. Louis, MO, USA) and Alfa-Aesar (Ward Hill, MA, USA) and were used as received, without further purification.

One-dimensional thin-layer chromatography (TLC) was performed on aluminum plates coated with silica gel 60F254 (Merck, Darmstadt, Germany) and visualized under UV light at 254 nm or by staining with a phosphomolybdic acid solution in ethanol.

All synthesized organic compounds were characterized by nuclear magnetic resonance (NMR) spectroscopy. ^1^H and ^13^C NMR spectra were recorded on a Bruker AVANCE III HD 400 MHz spectrometer (Karlsruhe, Germany) (^1^H: 400 MHz; ^13^C: 100 MHz). NMR samples were prepared in CDCl_3_. Chemical shifts (δ) are reported in parts per million (ppm).

Molecular recognition assays for MIPs and NIPs were performed using an Ohaus orbital shaker incubator (Parsippany, NJ, USA) at 37 °C and 300 rpm. The particles were separated by centrifugation at 4000 rpm for 15 min using a HERMLE Labortechnik Z307 centrifuge (Wehingen, Germany).

The calibration curve for donepezil·HCl quantification was constructed using a UV-Vis spectrophotometer (T80, PGI Instruments, Lutterworth, UK), with absorbance measured at 272 nm (the absorption wavelength of donepezil·HCl). The amount of donepezil·HCl was calculated based on the calibration curve.

Absorbance measurements for the release assays, metabolic viability tests, fluorescence emission spectra and antioxidant studies were performed using a Molecular Devices Spectra Max iD3 Multi-Mode Microplate Reader (San Jose, CA, USA).

The synthesized polymers were characterized by FTIR-ATR spectroscopy using a PerkinElmer Spectrum Two spectrometer (Seer Green, UK) equipped with an Attenuated Total Reflectance (ATR) accessory.

Morphological characterization of the synthesized polymers was performed using a scanning electron microscope with energy-dispersive X-ray spectroscopy (SEM-EDX), model Phenom (ThermoFisher Scientific, Waltham, MA, USA).

### 2.2. Synthesis of Functional Monomer Based on Quercetin

**Scheme 1:**
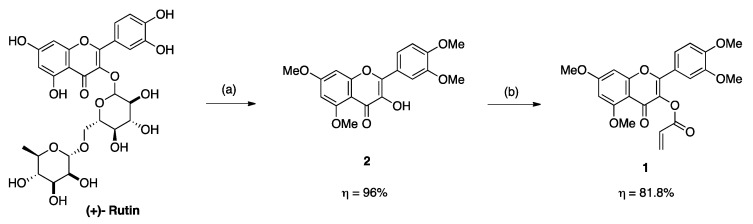
Synthetic pathway: (a) (i) K_2_CO_3_, MeI, DMF, RT, overnight; (ii) H_2_SO_4_ 4%, 115 °C, 3 h; (b) NEt_3_, DCM, acryloyl chloride, RT.

Scheme 1 depicts the synthetic strategy for the preparation of the functional monomer 3-acryloxy-3′,4′,5,7-tetramethylquercetin **1**.

#### 2.2.1. Synthesis of 3-Hydroxy-3′,4′,5,7-Tetramethylquercetin 2

In a 250 mL round-bottom flask fitted with a magnetic stir bar, (+)-rutin (5.0 g, 7.52 mmol, 1 eq.) and K_2_CO_3_ (5.6 g, 40.61 mmol, 5.4 eq.) were added and dissolved in 30 mL of DMF. Methyl iodide (CH_3_I, 2.54 mL, 40.61 mmol, 5.4 eq.) was then added under stirring and protected from light. The reaction mixture was stirred overnight. The DMF was removed by vacuum distillation at 60 °C. The resulting solid was carefully dissolved in 2% H_2_SO_4_ solution, and the mixture was refluxed at 115 °C for 3 h, resulting in the precipitation of a yellow solid. The mixture was allowed to cool, the solid was vacuum filtered, washed with distilled water, and dried under vacuum. Compound **2** was obtained as a yellow solid (2.59 g, yield = 96.0%).

**^1^H NMR (400 MHz, CDCl_3_) δ**: 3.90 (s, 3H, CH_3_), 3.94 (s, 3H, CH_3_), 3.95 (s, 3H, CH_3_) 3.97 (s, 3H, CH_3_), 6.31 (d, 1H, J = 2.2 Hz, CH), 6.5 (d, 1H, J = 2.2 Hz, CH), 6.99 (d, 1H, J = 9 Hz, CH), 7.41 (s, 1H, OH), 7.75–7.78 (m, 2H, 2xCH) ppm.

**^13^C APT NMR (100 MHz, CDCl_3_) δ**: 55.9, 56.0, 56.1, 56.5, 92.5, 95.7, 106.2, 110.3, 110.9, 120.7, 123.8, 137.6, 142.1, 148.8, 150.3, 158.9, 160.5, 164.4, 171.9 ppm.

#### 2.2.2. Synthesis of 3-Acryloxy-3′,4′,5,7-Tetramethylquercetin 1

The esterification of compound **2** was carried out by adding triethylamine (0.82 mL, 5.86 mmol, 3.0 eq.) to a solution of compound **2** (0.70 g, 1.95 mmol) in 20 mL of dichloromethane (DCM). The mixture was stirred for 30 min, then cooled in an ice bath, followed by the dropwise addition of acryloyl chloride (0.47 mL, 5.86 mmol, 3.0 eq.). The reaction mixture was stirred at room temperature for 5 h. The product was extracted with saturated NaHCO_3_ solution (3 × 50.0 mL). The organic phase was dried over anhydrous Na_2_SO_4_, filtered and the solvent was removed under reduced pressure. The crude product, obtained as a brown solid, was purified by column chromatography on silica gel using a 1:9 mixture of hexane and ethyl acetate as the eluent. Compound **1** was obtained as a white solid (0.657 g, yield = 81.8%).

**^1^H NMR (400 MHz, CDCl_3_) δ**: 3.90 (s, 3H, CH_3_), 3.90 (s, 3H, CH_3_), 3.92 (s, 6H, CH_3_), 3.93 (s, 6H, CH_3_), 6.02 (dd, 1H, *J =* 1.3, 10.4 Hz, CH), 6.36 (d, 1H, *J* = 2.4 Hz, CH), 6.37 (dd, 1H, *J* = 10.5, 17.3 Hz, CH), 6.52 (d, 1H, *J =* 2.3 Hz, CH), 6.63 (dd, 1H, *J =* 1.3, 17.3 Hz, CH), 6.94 (d, 1H, *J =* 8.6 Hz, CH), 7.37 (d, 1H, *J =* 2.1 Hz, CH), 7.48 (dd, 1H, *J =* 2.1, 8.5 Hz, CH) ppm.

**^13^C APT NMR (100 MHz, CDCl_3_) δ**: 55.9, 56.1, 56.1, 56.4, 92.7, 96.2, 108.9, 110.9, 111.0, 121.8, 122.4, 127.3, 133.4, 133.6, 148.9, 151.3, 153.4, 159.2, 161.3, 163.3, 164.4, 170.6 ppm.

### 2.3. Synthesis of Molecularly Imprinted Polymers Selective to Donepezil

For the synthesis of MIPs, 0.03 g (0.0791 mmol, 1 eq.) of donepezil·HCl was dissolved in 5 mL of acetonitrile (ACN), 1 mL of DMSO, and 1 mL of CHCl_3_ in a screw-cap reaction tube. The functional monomers acrylic acid and monomer **1** were added, and the mixture was subjected to pre-polymerization in an ultrasonic bath for 15 min, to remove dissolved oxygen, followed by nitrogen purging for 5 min. Then, the crosslinker was added: either EGDMA (14 eq., 1.1 mmol, 0.21 mL), or TMPTA (14 eq., 1.1 mmol, 0.35 mL), along with the radical initiator AIBN (2.3 eq., 0.180 mmol, 0.0299 g). The tube was sealed, and reaction mixture was then heated at 70 °C for 24 h. For each MIP synthesized, the specific proportions of all monomers and crosslinkers are detailed in Table 1.

After polymerization, the resulting polymers were washed by Soxhlet extraction using a MeOH:AcOH mixture (9:1) for 24 h, followed by MeOH for another 24 h. The polymers were then dried under vacuum. Simultaneously, the corresponding NIP (non-imprinted polymer) was synthesized using the same procedure, but in the absence of donepezil·HCl. Using this procedure, four MIPs and their respective NIPs were synthesized, as summarized in Table 1.

### 2.4. Molecular Recognition Assays

#### 2.4.1. Preparation of Donepezil and MIP/NIP Solutions at Different Concentrations

Aqueous solutions of donepezil·HCl at concentrations of 6.25, 12.5, 25.0, 50.0, and 90.0 mg/L were prepared by successive dilutions from a stock solution of 180 mg/L for the construction of the calibration curve. These solutions were analyzed by UV-Vis spectroscopy at 272 nm.

#### 2.4.2. Determination of Adsorption Capacity

For the previously prepared MIPs and NIPs, solutions were prepared by dispersing 5.0 mg of polymer in 5 mL of donepezil·HCl solution at concentration of 90.0 mg/L. The samples were incubated at 37 °C under orbital shaking at 300 rpm for 30 min. After incubation, the solutions were centrifuged at 4000 rpm for 20 min, and the supernatants were decanted and analyzed by UV-Vis spectroscopy at 272 nm. All assays were performed in triplicate. The adsorption capacity (q_e_) and retention rate (RR, %), were determined according to Equations (1) and (2) [34]. The adsorption capacity was calculated based on the initial concentration (C_0_, mg/L), the equilibrium concentration was that which remained in the solution after equilibrium was reached (Cₑ, mg/L), the solution volume (V, L), and the polymer mass (m, g):

(1) $$q_{e}=\frac{(C_{0}-C_{e})\times V}{m}$$

The retention rate (RR, %) represents the percentage of donepezil·HCl retained by the polymer and was determined by:

(2) $$RR\left(\%\right)=\frac{{(C_{0}-C}_{e})}{C_{0}}$$

These parameters provide insight into the affinity and selectivity of the imprinted polymer toward donepezil·HCl, allowing comparison between the molecularly imprinted (MIP) and non-imprinted (NIP) materials.

#### 2.4.3. Adsorption Isotherms of Donepezil

For the adsorption isotherm studies, 5 mg of each polymers MIP-4 and NIP-4 was added into 15 mL conical centrifuge tubes with 5 mL aqueous solution of donepezil·HCl at concentrations of 1.56, 3.13, 6.25, 12.5, 25, 50, 75, 90, 100, 145 mg/L, respectively. The tubes were incubated at 37 °C under orbital shaking at 200 rpm for 30 min, and centrifuged at 4000 rpm for 20 min. The supernatants were separated, and their donepezil·HCl concentrations were determined (in triplicate) by spectrophotometry UV-Vis at 272 nm. The amounts of donepezil·HCl adsorbed (q_e_) were calculated according to Equation (1). The adsorption results were analysed with Langmuir and Freundlich equations, according to Equations (3) and (4), respectively [34]:

(3) $$\frac{C_{e}}{q_{e}}=\;\frac{1}{q_{\mathrm{mL}}K_{L}}+\frac{C_{e}}{q_{\mathrm{mL}}}$$

(4) $$\log q_{e}=\log K_{F\;}+\frac{1}{n_{F}}\log C_{e}$$

where q_mL_ (mg/g) is the monolayer capacity obtained from the Langmuir plots, K_L_ (L/mg) is Langmuir’s constant, K_F_ ((mg/g)/(mg/L)^1/nF^) and n_F_ are the parameters of the Freundlich equation.

### 2.5. Cell Viability Assays

#### 2.5.1. Maintenance of MCF-7 Cells

The human breast carcinoma MCF-7 cell line was used to access MIPs and NIPs cytotoxicity. MCF-7 cells were grown in EMEM media supplemented with 10% FBS. For the assays, cells were seeded into 96-well microplates at a density of approximately 5000 cells per well and incubated for 24 h in a CO_2_ incubator to allow cell adhesion and stabilization before treatment.

#### 2.5.2. Preparation of MIP/NIP Solutions

Serial dilutions of MIP-3, NIP-3, MIP-4, NIP-4, were prepared at concentrations of 3.3, 10, 30, and 90 μg/mL of culture media. Donepezil·HCl solutions were prepared at 2.2, 6.6, 20, and 60 μg/mL.

#### 2.5.3. Exposure of Cells to MIPs and NIPs

Cells were exposed in quadruplicate to 100 μL of each formulation (MIP-3, MIP-4, and their corresponding NIPs) at the tested concentrations. Each plate included negative controls (cells in culture medium) and positive controls (cells exposed to 5% SDS solution). Incubation of the cells was conducted over a 24 h period in a CO_2_ incubator.

#### 2.5.4. WST-8 Assay

Cell metabolic viability was determined using the WST-8 Cell Proliferation Assay Kit (Cayman Chemical Company, Ann Arbor, MI, USA), following the reduction of the WST-8 tetrazolium salt by the cellular dehydrogenases, to formazan product, according to the supplier instruction.

#### 2.5.5. Fluorescein Diacetate (FDA) Exposure

A fluorescein diacetate (FDA) working solution (2 μg/mL) was prepared in phosphate-buffered saline (PBS, 100 μM, pH 7). For the FDA viability assay, all the culture media were removed, and the cells were washed once with 100 μL of PBS (100 μM, pH 7). Subsequently, 100 μL of the FDA working solution was added to each well, and the plate was incubated for 5 min at 37 °C. After FDA loading, the solution was removed, and the wells were washed again with PBS, removing the FDA that was not incorporated in the cells and eventually the fluorescein leaked by non-viable cells (with damaged membrane integrity). After this final wash, 150 μL of 0.5% Triton X-100 solution was added to each well. The plate was incubated for 5 min at 37 °C in a CO_2_ incubator. Subsequently, 50 μL of the resulting lysate was transferred to an empty opaque 96-well microplate, and the fluorescence intensity of fluorescein was recorded at λ_ex_ = 485 nm and λ_em_ = 535 nm using a microplate fluorimeter.

### 2.6. In Vitro Drug Release

#### 2.6.1. Preparation of Donepezil Solutions at Different pH Values (3.0, 5.5, and 7.0)

The in vitro release study was performed in three media with different pH values: 3.0, 5.5, and 7.0. The following buffer solutions were prepared: 100 mM sodium citrate buffer (pH 3.0), 100 mM phosphate-buffered saline (PBS, pH 5.5), and 100 mM PBS (pH 7.0). Calibration curves were prepared for each medium using donepezil·HCl solutions at an initial concentration of 360.0 mg/L in each buffer. Serial dilutions were then performed to obtain standard solutions with concentrations between 3.1 and 180.0 mg/L.

#### 2.6.2. Loading of Polymers with Donepezil

Accurately weighed 25.0 mg of MIP or NIP was transferred into a 2 mL microtube, followed by the addition of 1.7 mL of donepezil·HCl aqueous solution (400 mg/L) prepared in the corresponding buffer (pH 3.0, 5.5, or 7.0). The mixture was incubated at 37 °C under agitation for 15 h. After incubation, the suspension was centrifuged, and the supernatant was decanted and reserved. The MIP was washed with 500 μL of water, centrifuged again, and the wash solution was also collected. The amount of donepezil·HCl in the supernatant and the wash solutions was determined by UV–Vis spectroscopy using the corresponding calibration curve.

The amount of donepezil·HCl immobilized within the MIP and NIP was calculated as the difference between the initial and final (supernatant) concentrations. The loaded MIP and NIP were then stored for subsequent in vitro release studies.

#### 2.6.3. In Vitro Release of Donepezil·HCl

To each microtube containing MIP or NIP loaded with donepezil·HCl, 1.7 mL of the respective release medium (100 mM sodium citrate buffer, pH 3.0; PBS, pH 5.5; or PBS, pH 7.0) was added. The samples were incubated at 37 °C under agitation for 30 min. After incubation, the samples were centrifuged for 2 min, and the supernatant was collected for UV–Vis analysis at 272 nm (measured in triplicate). Fresh buffer (1.7 mL) was then added to the sedimented MIP/NIP, and incubation was continued for 1 h. The same procedure of centrifugation, collection, and analysis of the supernatant was repeated periodically over a total period of 260 h (10 days). All experiments were performed in duplicate for each pH condition. Assuming no burst effect or lag time, the Korsmeyer-Peppas model is defined by Equation (5) [37]:

(5) $$\frac{Q_{t}}{Q_{\infty }}=k\times t^{n}$$

where k is the diffusion constant accounting for the geometric and structural characteristics of the dosage form, Q_t_ is the fraction of drug released at time t, and Q_∞_ is the total amount released. The exponent n describes the release mechanism. Values of n, k, and the correlation coefficients (R^2^) were obtained for the three buffer (pH 3, 5.5, 7) conditions.

### 2.7. In Vitro Cholinesterases Inhibitory Assays

Evaluation of the Inhibitory Activity of the Materials against eeAChE and eqBuChE. In a 96-well microplate, 20 µL of different concentrations of donepezil·HCl, 55 µL of Tris-HCl buffer (0.05 M, pH 8.0), 125 µL of DTNB (3.0 mM or 1.6 mM), and 25 µL of *Electrophorus electricus* acetylcholinesterase (*ee*AChE, 0.3 U/mL) or equine butyrylcholinesterase (eqBuChE, 0.3 U/mL) were added to each well. The mixture was incubated for 15 min at 37 °C. Subsequently, 25 µL of ATCI (15 mM) or BTCI (8.0 mM) was added to each well, followed by another incubation for 15 min at 37 °C.

Finally, the absorbance was measured at 412 nm and 25 °C. All assays were performed in triplicate.

### 2.8. Ferric Reducing Antioxidant Power (FRAP) Assay

The antioxidant activity of the samples was determined, according to the method described by Benzie and Strain (1996), with small modifications [38]. The FRAP reagent was freshly prepared by mixing 100 mL of sodium acetate buffer (300 mM, pH 5.0), 10 mL of 2,4,6-tripyridyl-*s*-triazine (TPTZ) solution (10 mM in 40 mM HCl), and 10 mL of ferric chloride hexahydrate (20 mM in distilled water). For the assay, 20 µL of each sample solution or Trolox standard was added to a 96-well microplate, followed by the addition of 200 µL of the FRAP reagent. The reaction mixture was incubated at 37 °C, and absorbance was measured at 593 nm after 2 min. Trolox solutions ranging from 0.0156 to 16 mg/mL were used to construct the calibration curve, and results were expressed as mg of Trolox equivalents per mL of sample (mg TE/mL). The tested samples included QUER at concentrations of 0.625, 1.25, and 2.5 µg/mL; monomer **1** at 0.205, 0.41, and 0.82 µg/mL; MIP-4 at 3.3, 10, and 30 µg/mL; and NIP-4 2-2 at 3.3, 10, and 30 µg/mL.

### 2.9. Statistical Analysis

ANOVA test was used to test statistical significance. A *p*-value lower than 0.05 was considered as statistically significant.

## 3. Results and Discussion

### 3.1. Synthesis of Functional Monomer Derived from Quercetin

The first step involved the preparation of the functional monomer derived from quercetin for the synthesis of MIPs selective to Donepezil and respective NIPs. The selected derivative was 3-acryloxy-3′,4′,5,7-tetramethylquercetin **1** (Scheme 1), in which the hydroxyl groups are protected with methyl groups. This modification was intended to furnish a certain degree of hydrophobicity to the MIPs, facilitating their ability to permeate the blood–brain barrier.

The synthesis of the functional monomer 3-acryloxy-3′,4′,5,7-tetramethylquercetin **1** was carried out in two steps, according to Scheme 1. The first involved the preparation of the intermediate 3-hydroxy-3′,4′,5,7-tetramethylquercetin **2**, and the second consisted of an esterification reaction. For the synthesis of 3-hydroxy-3′,4′,5,7-tetramethylquercetin **2**, the method described by Kajjout and Roland [39,40,41,42] was followed. It involved the methylation of (+)-rutin through the deprotonation of hydroxyl groups using K_2_CO_3_ as the base, followed by a nucleophilic substitution with iodomethane (MeI) in DMF, thus forming methylated quercetin **2** (Scheme 1a). The mechanism involved is a bimolecular nucleophilic substitution (S_N_2). Subsequently, the *O*-rutinoside group was hydrolyzed under acidic conditions using an acidic methanolic solution, affording 3-hydroxy-3′,4′,5,7-tetramethylquercetin **2** in 96% yield. The second step consisted of the esterification of 3-hydroxy-3′,4′,5,7-tetramethylquercetin **2**, which proceeded via an acyl nucleophilic substitution mechanism. Triethylamine (NEt_3_) was used as a base to deprotonate the hydroxyl group, generating an alkoxide that subsequently attacked acryloyl chloride, affording the desired 3-acryloxy-3′,4′,5,7-tetramethylquercetin **1** with a yield of 78% (Scheme 1b).

The structure of the quercetin derivatives **1** and **2** were confirmed by ^1^H and ^13^C NMR spectroscopy (Figures S1–S4). The structure of intermediate **2** has been previously reported in the literature, and its ^1^H and ^13^C APT NMR spectra (Figures S1 and S2) are in agreement with the data described in the literature [40,41,42]. The structure of the functional monomer 3-acryloxy-3′,4′,5,7-tetramethylquercetin **1** was confirmed by ^1^H and ^13^C APT NMR analyses (Figures S3 and S4). The protons of the four methoxy groups (–OCH_3_) appear as four singlets within the chemical shift range of δ 3.90–3.94 ppm. The five aromatic protons of the quercetin scaffold are observed as four doublets at δ 6.36 (J = 2.4 Hz), δ 6.52 (J = 2.3 Hz), δ 6.94 (J = 8.6 Hz), and δ 7.37 ppm (J = 2.0 Hz), together with one doublet of doublets at δ 7.48 ppm (J = 2.1 and 8.5 Hz).

The three vinyl protons of the acrylate moiety appear as three doublets of doublets: the −CH_2_ protons resonate at δ 6.02 ppm (J = 1.3 and 10.4 Hz) and δ 6.63 ppm (J = 1.3 and 17.3 Hz), while the –CH proton is observed at δ 6.37 ppm (J = 10.5 and 17.3 Hz).

The ^13^C APT NMR spectrum (Figure S4) further corroborates the proposed structure, showing characteristic aromatic –CH signals of the quercetin scaffold at δ 92.7, 96.2, 110.9, 111.0, and 121.8 ppm, as well as the vinyl CH and CH_2_ signals at δ 127.3 and 133.4 ppm, respectively. The four methoxy carbon signals appear at δ 55.9, 56.1, 56.1, and 56.4 ppm, in agreement with the assigned structure.

### 3.2. Synthesis of the Molecularly Imprinted Polymer Selective to Donepezil

**Figure 1:**
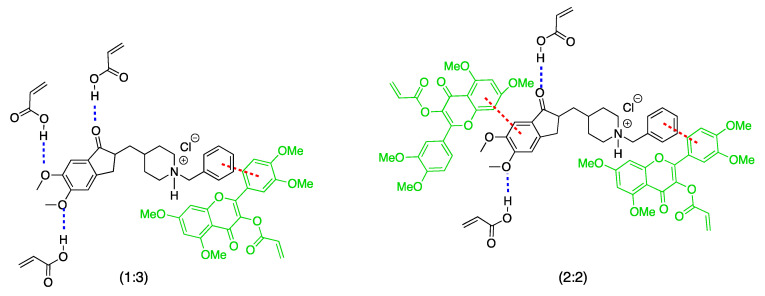
Hypothetical pre-polymerization complexes formed between the functional monomers, namely acrylic acid and monomer **1**, and donepezil·HCl via hydrogen bonding (blue dashed lines) and π–π stacking (red dashed lines).

**Scheme 2:**
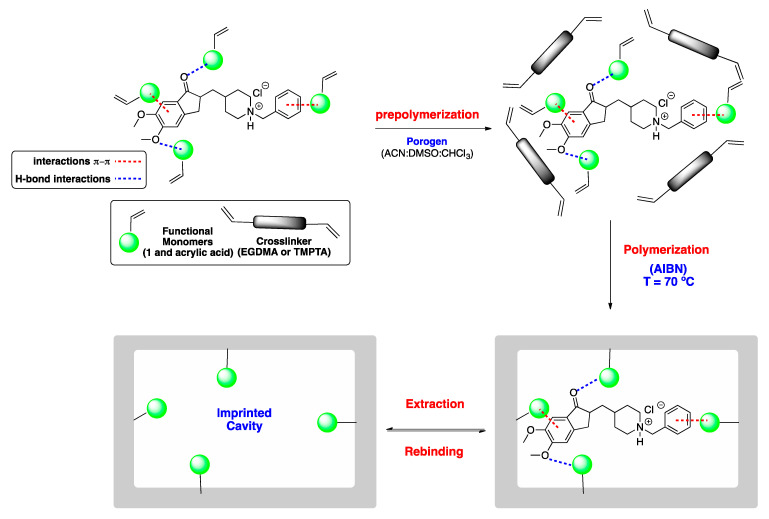
Generic scheme for the synthesis of molecularly imprinted polymers (MIPs) selective for donepezil·HCl.

Four MIPs selective for donepezil·HCl, designated by MIP-1, 2, 3 and 4, along with their respective NIPs, NIP-1, 2, 3 and 4, were successfully synthesized via bulk radical polymerization using thermal initiation with the radical initiator AIBN, methodology already established in our group, as illustrated in Scheme 2 [23,24,25,43]. With the aim of developing a selective polymer for donepezil·HCl based on renewable natural product derivatives, a functional monomer derived from quercetin (monomer **1**), previously synthesized, was combined with acrylic acid. Monomer **1**, possesses multiple interaction sites capable of engaging in hydrogen bonding, dipole–dipole interactions, hydrophobic interactions and especially π–π stacking interactions with the template molecule, donepezil·HCl. Acrylic acid, on the other hand, acts as a strong hydrogen bond donor and also exhibits pH-responsive behaviour. These non-covalent interactions are crucial for the formation of stable pre-polymerization complexes, thereby contributing to the high affinity and selectivity of the resulting imprinted binding sites (Figure 1). The effect of two crosslinkers, EGDMA and TMPTA, was also investigated, since crosslinkers play an important role in stabilizing the complex formed during the pre-polymerization step [23]. To investigate the influence of the monomer ratio and type of crosslinker on the performance of the imprinted polymers, four different MIP formulations were developed. MIP-1 and MIP-3 were synthesized using a 1:3 molar ratio of monomer **1** to acrylic acid. The difference between these two lies in the crosslinking agent: EGDMA was used in MIP-1, whereas TMPTA was employed in MIP-3. The pre-polymerization complex formed between donepezil·HCl and the functional monomers, tetramethylquercetin (monomer **1**) and acrylic acid, is primarily stabilized by hydrogen bonding between the hydroxyl groups of acrylic acid and π–π interactions between the aromatic rings of tetramethylquercetin and the benzyl moiety of donepezil. Although dipole–dipole and hydrophobic interactions are not illustrated in Figure 1, their potential contribution to stabilization cannot be entirely disregarded.

On the other hand, MIP-2 and MIP-4 were prepared using a 2:2 molar ratio of monomer **1** to acrylic acid, with MIP-2 crosslinked by EGDMA and MIP-4 by TMPTA. In these systems, π–π, dipole–dipole, and hydrophobic interactions are expected to dominate over hydrogen bonding in stabilizing complexes between the functional monomers and the template (Figure 1), as both the tetramethylquercetin scaffold and the indanone/phenyl moieties of donepezil are aromatic and nonpolar. The planar π-systems facilitate close ring stacking, enhancing stabilization primarily through dispersion forces, which act more extensively and less directionally than hydrogen bonds.

These variations in composition were designed to modulate the network rigidity, porosity, and definition of the imprinted cavities.

### 3.3. Physicochemical Characterization—Chemical Composition (FTIR-ATR)

**Figure 2:**
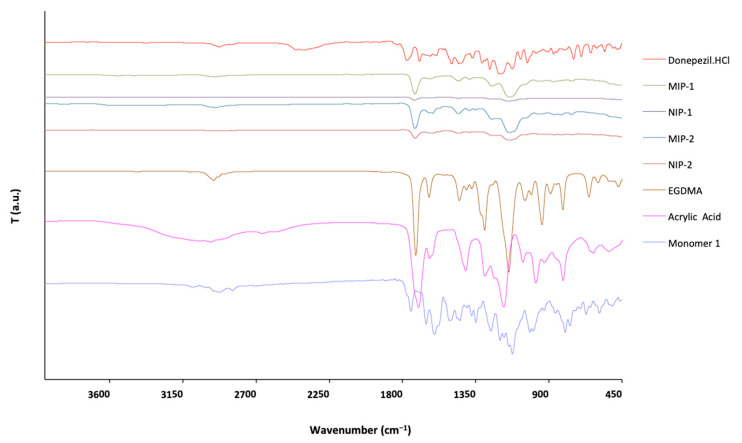
FTIR-ATR spectra of donepezil·HCl, MIP-1 and MIP-2, and their corresponding NIPs; and the starting materials.

**Figure 3:**
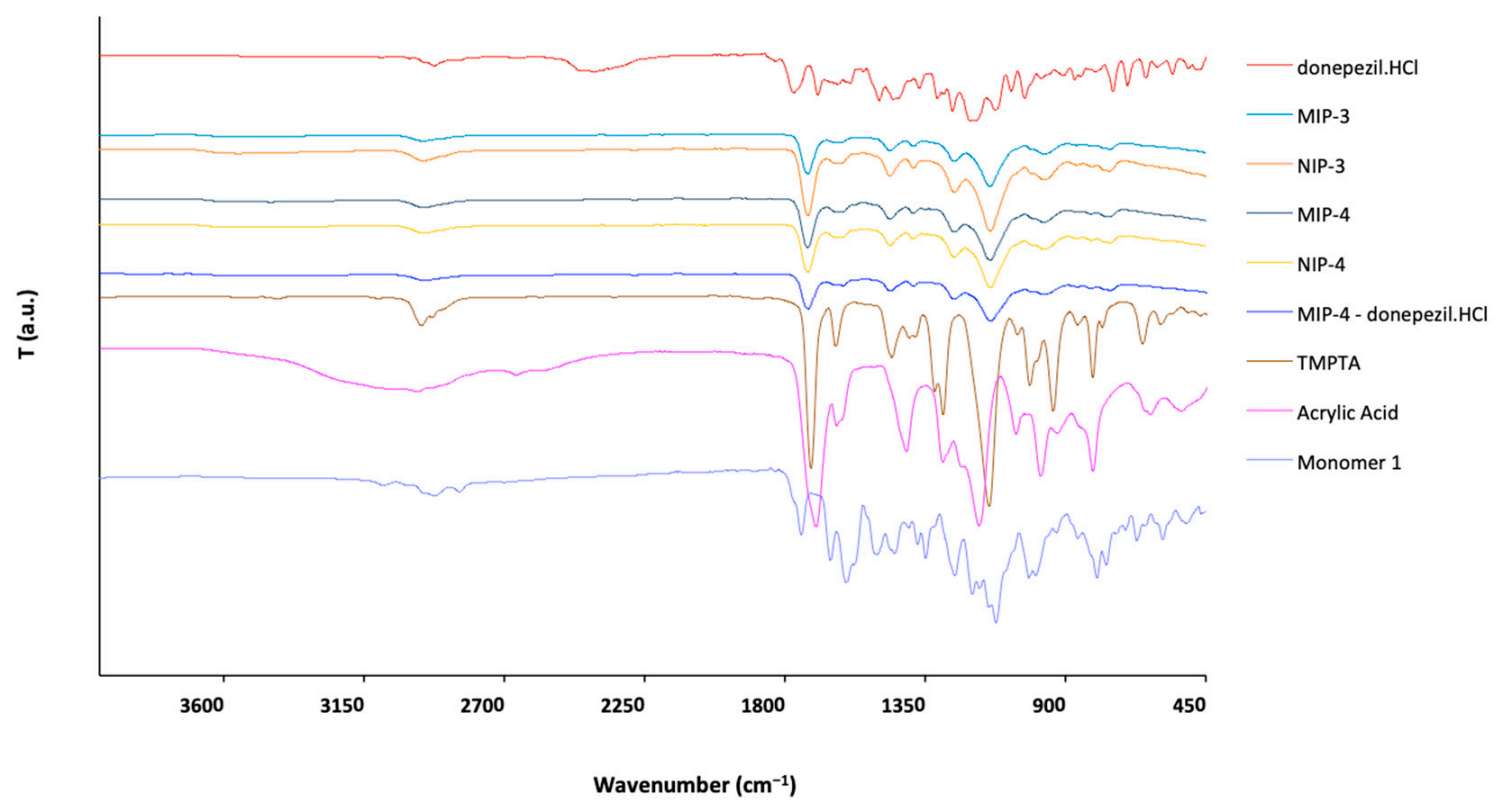
FTIR-ATR spectra of donepezil·HCl, MIP-3 and MIP-4, and their corresponding NIPs; loaded MIP-4 and the starting materials.

The four MIPs and their corresponding NIPs prepared for donepezil were characterized in terms of chemical composition by Fourier-transform infrared spectroscopy (FTIR-ATR). Figure 2 presents the spectra of MIPs 1 and 2 and NIPs 1 and 2, together with those of the starting materials (monomer **1**, acrylic acid, EGDMA, and donepezil·HCl). Figure 3 shows the spectra of MIPs 3 and 4, their respective NIPs, loaded MIP-4, and the corresponding starting materials, including TMPTA. The FTIR spectrum of monomer **1** (quercetin derivative) shows characteristic bands at 2972 and 2882 cm^−1^ (aromatic and aliphatic C–H and C–O–CH_3_ stretching), 1756 cm^−1^ (ester C=O stretching), 1660 cm^−1^ (C=C stretching), 1612 cm^−1^ (C=O stretching of the quercetin framework), 1520 cm^−1^ (aromatic C=C stretching), 1461 cm^−1^ (C–H bend), 1376–1266 cm^−1^ (C=C–O–C ether stretching) and in the 1206–1148 cm^−1^ region (C–O–C stretching) and at 808 cm^−1^ (=C–H stretching), confirming its functionalized flavonoid structure [41]. Acrylic acid exhibits the expected absorptions bands at 3068 cm^−1^ (broad O–H and C–H stretching), 1734 cm^−1^ (C=O stretching), 1666 cm^−1^ (C=C stretching), in the 1206–1148 cm^−1^ region (C–O–C stretching) and 818 cm^−1^ (=C–H stretching). The crosslinkers EGDMA and TMPTA display similar spectral features, with characteristic bands at approximately 2988 cm^−1^ (aliphatic C–H stretching), 1720 cm^−1^ (ester C=O stretching), 1644 cm^−1^ (C=C stretching) and in the 1206–1148 cm^−1^ region (C–O–C stretching) and 818 cm^−1^ (=C–H stretching) [36].

The FTIR spectra of all polymers are highly similar, confirming successful polymerization. In particular, the disappearance of the vinyl =C–H stretching band around 818 cm^−1^ indicates effective consumption of the double bonds during polymer formation. The presence of ester functionalities in all polymers is confirmed by the intense C=O stretching band at approximately 1740 cm^−1^ and by the C–O stretching bands observed between 1160 and 1278 cm^−1^, originating from both monomers and crosslinkers. Aliphatic C–H bending and stretching vibrations at around 1416 and 2982 cm^−1^ further confirm the formation of polymeric networks. A weak O–H stretching band around 3602 cm^−1^ indicates the incorporation of acrylic acid units, while a low-intensity and broad band near 1650 cm^−1^, attributed to C=C and C=O (quercetin framework) stretching vibrations, suggests the presence of the quercetin-derived monomer **1** within the polymer matrix. Comparison of the FTIR spectra of MIPs and their corresponding NIPs reveals no significant differences, indicating identical chemical composition and confirming that the imprinting process does not alter the polymer backbone.

**Figure 4:**
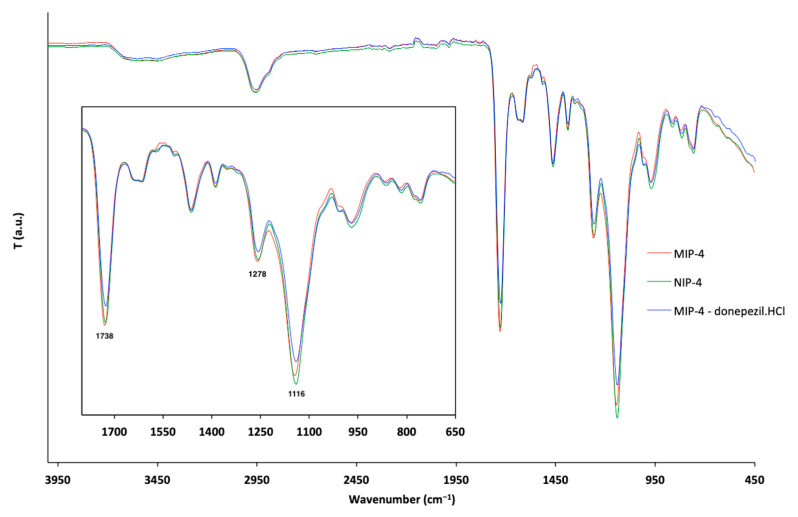
Comparison of the FTIR-ATR spectra of MIP-4, loaded MIP-4 (MIP– 4-donepezil·HCl), and NIP-4.

Comparison of the FTIR spectra (Figure 4) of MIP-4, loaded MIP-4, and NIP-4 reveals subtle variations in the absorption bands at approximately 1738, 1116, and 1278 cm^−1^, which are attributed to the C=O and C–O stretching vibrations of ester groups originating from the monomers and crosslinker. In the spectrum of loaded MIP-4, these bands exhibit slightly reduced intensities relative to those of MIP-4 and NIP-4. Such changes, observed suggest the establishment of interactions between donepezil·HCl and the polymer matrix and are consistent with the incorporation of the drug within the imprinted polymer network.

Additionally, MIP-4, NIP-4, and MIP-4 loaded with donepezil·HCl (loading of 364.68 µg) were subjected to extraction using a MeOH/acetic acid mixture (6:4, *v*/*v*), according to the procedure described in the Supplementary Materials, with the aim of evaluating the efficiency of template removal after polymer synthesis and validating the extraction method using the loaded MIP-4 sample. The extraction process involved repeated washing cycles under ultrasonic bath, with the choice of this solvent system based on its ability to disrupt hydrogen-bonding interactions established between the functional monomers and donepezil·HCl, thereby facilitating its removal. The obtained extracts were quantitatively analyzed by UV–Vis spectroscopy in the 230–400 nm range (Figure S5). No characteristic absorption bands of donepezil·HCl were detected in extracts from unloaded MIP-4 and NIP-4 samples, whereas the encapsulated drug was completely extracted from the loaded MIP-4 under the same conditions. Nevertheless, the presence of trace residual amounts in the polymer matrix cannot be entirely excluded.

### 3.4. Fluorescence of Quercetin-Derived Monomer 1 and Polymers

Quercetin and its derivatives are also well known for their intrinsic fluorescence properties, attributed to their extended π-conjugated flavonol system, which facilitates efficient light absorption and emission [44]. In aqueous solution, native quercetin, which bears free phenolic OH groups, exhibits weak fluorescence with an excitation maximum around 370 nm and an emission band centered at ~532 nm as reported by Sahu et al. The photophysical behavior is strongly influenced by hydrogen bonding and solvent polarity [44].

**Figure 5:**
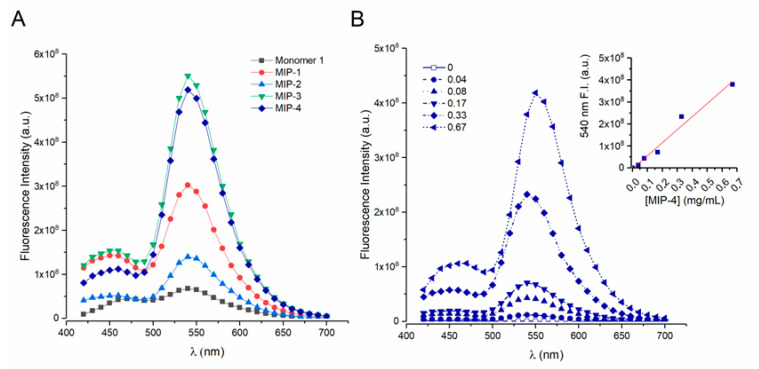
(**A**) Fluorescence emission spectra of monomer **1** and MIPs 1–4 in water (λ_exc_ = 380 nm); (**B**) Fluorescence emission spectra at different concentrations of MIP-4 in water (λ_exc_ = 380 nm) and inlet graphic representing 540 nm emission vs. MIP-4 concentration.

In the present study, the quercetin-derived monomer **1**, in which phenolic OH groups are methylated and the C-3 OH is esterified with an acrylate moiety, showed an emission maximum at ~540 nm when excited at 380 nm, indicating that the conjugated chromophore remains after functionalization (Figure 5A). The small bathochromic shift in both excitation and emission wavelengths relative to native quercetin is likely due to chemical modification of the hydroxyl groups, which alters the electronic distribution of the flavonol chromophore and reduces hydrogen-bonding interactions with water. The polymers were analyzed under the same aqueous conditions (λ_exc_ = 380 nm). As shown in Figure 5A, all polymeric materials exhibit emission bands similar to that of monomer **1**, indicating that the quercetin-derived fluorophore retains its intrinsic fluorescence after incorporation into the polymer matrix. A detailed fluorescence analysis of MIP-4 was conducted in water, with emission measurements performed at an excitation of 380 nm and a corresponding emission maximum at 540 nm (Figure 5B). Furthermore, the fluorescence intensity of MIP-4 increased proportionally with concentration across the range of 0.04 to 0.67 mg/mL (Figure 5B, inlet). Fluorescence characteristics of MIP-4 were also verified not only in water, but also in solution buffered at pH 3.3, 7 and 9. These findings suggest that the quercetin-derived units within the polymer structure act as intrinsic fluorescent probes. This feature is particularly advantageous for drug delivery systems, as it enables real-time tracking of the polymer without requiring external fluorescent labels, which may interfere with the material’s properties or biocompatibility. The inherent fluorescence can thus be exploited to monitor polymer distribution, cellular uptake, and drug release profiles in biological environments, enhancing the understanding and control of the therapeutic process [45].

### 3.5. Recognition Capacity of the Polymers

The recognition ability of the synthesized polymers towards donepezil·HCl in aqueous solution (pH = 5.88) was evaluated through adsorption capacity (q_e_) and retention rate (RR, %), with results summarized in Table 2. The polymers MIP-1 and MIP-3, prepared using a mixture of monomer **1** and acrylic acid in a 1:3 ratio, showed very similar values for both q_e_ and RR when compared with their corresponding NIPs, indicating low imprinting efficiency and poor selectivity toward donepezil·HCl in these systems. However, MIP-3, which was synthesized using TMPTA as the crosslinker, exhibited higher values of 34.54 ± 1.40 mg/g for q_e_, and 38.37 ± 1.56% for RR, suggesting that the nature of the crosslinker may have a minor influence on the adsorption performance. This behavior may be attributed to the high crosslinking density of TMPTA-based polymers, which, despite being commonly associated with reduced overall porosity, in the present system appear to generate a controlled and functionally optimized porous architecture that favors molecular diffusion and selective recognition.

Regarding the putative mechanism of adsorption, the low selectivity observed in water can be explained by the fact that the putative hydrogen bonding between donepezil·HCl (pKa = 8.9 [35]) and the acrylic polymer (pKa ≈ 4.5 [46]) is negligible (Figure 1), as the template is fully protonated while the polymer is deprotonated. Consequently, adsorption is primarily governed by noncovalent interactions, including electrostatic interactions between the –COO^−^ groups of the polymer and –NH^+^ of donepezil, as well as π–π stacking, dipole–dipole and hydrophobic interactions. While electrostatic and dipole–dipole contributions may be partially weakened in aqueous solution, π–π and hydrophobic interactions are expected to remain largely effective.

On the other hand, MIP-2 and MIP-4 were synthesized using a 2:2 ratio of the same functional monomers. Only MIP-4 demonstrated a moderate affinity toward donepezil·HCl, and its q_e_ and RR values were significantly higher than those of MIP-2. MIP-2 and its corresponding NIP, similar to MIP-1 and NIP-1, were both prepared using EGDMA as the crosslinker and displayed slightly lower adsorption performance compared with the MIP/NIP-3 and MIP/NIP-4 polymers, which were synthesized with TMPTA. Interestingly, MIP-4 exhibited a q_e_ of 32.90 ± 0.5 mg/g and a RR of 36.56%, which is slightly higher than the respective NIP-4 (27.50 ± 0.40 mg/g and 30.56%). This modest difference indicates the potential presence of selective binding sites. Such selectivity likely arises from π–π stacking, dipole–dipole and hydrophobic interactions provided by the tetramethylquercetin monomer **1** and hydrogen bonding from acrylic acid during pre-polymerisation (Figure 1), combined with the structural rigidity imparted by TMPTA, which may promote more defined binding cavities and increase the number of hydrophobic sites within the polymer network, potentially enhancing the adsorption of donepezil·HCl compared to the EGDMA-based polymer.

In the adsorption mechanism, hydrogen bonding is negligible due to the full deprotonation of the polymer (pKa ≈ 4.5) and protonation of donepezil·HCl (pKa = 8.9), electrostatic interactions are also partially weakened in water because the charges are strongly solvated. Under these conditions, adsorption is primarily driven by π–π and hydrophobic interactions. The largely nonpolar, aromatic tetramethylquercetin-based monomer facilitates these interactions with donepezil·HCl, contributing to the modestly higher adsorption observed for MIP-4 compared to NIP-4, likely arising from π–π interactions within partially non-specific cavities.

Statistical analysis confirmed significant differences between MIPs 3 and 4 and their respective NIPs, whereas no significant differences were observed for MIPs 1 and 2, suggesting that the crosslinker contributes to the molecular recognition process, which, however, is modest.

**Figure 6:**
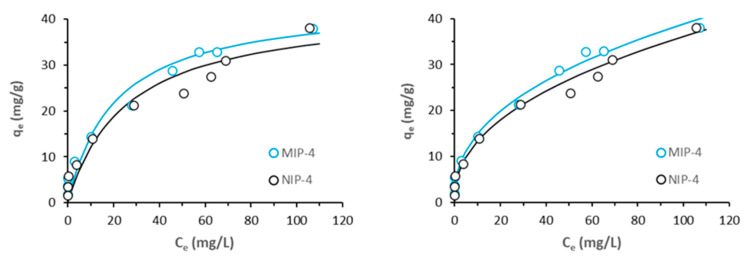
Adsorption isotherms at 37 °C of Donepezil·HCl on MIP-4 and NIP-4. The circles correspond to experimental points and the lines correspond to the curves calculated with Langmuir (**left**) and Freundlich (**right**) equations.

The adsorption behaviour of MIP-4 and NIP-4 towards donepezil·HCl was evaluated through adsorption isotherm studies. The experimental results are shown in Figure 6. At the highest used concentration of donepezil·HCl, both materials reached similar adsorption capacities, with q_e_ values of approximately 38 mg/g. However, the MIP-4 exhibited slightly higher q_e_ than NIP-4 for most points of the isotherm, and a regular and typical isotherm profile, whereas the isotherm of NIP-4 suggests the presence of heterogeneous adsorption sites in the NIP, likely associated with non-specific interactions and structural irregularities in the polymer matrix, in contrast to the more uniform and selective binding cavities of the MIP.

Adsorption data were analysed using Langmuir and Freundlich equations in the linear form (Equations (3) and (4)), to better characterize the adsorption behaviour. The results are presented in Table 3 and the curves calculated using these values are the lines in Figure 6.

The Langmuir model, which assumes monolayer adsorption on homogeneous sites, fits better for MIP-4 (R^2^ = 0.975) than for NIP-4 (R^2^ = 0.929), consistent with the presence of specific, uniform binding sites generated by molecular imprinting. MIP-4 also exhibited a slightly higher Langmuir adsorption capacity at saturation (q_mL_ = 43.86 mg/g) than NIP-4 (q_mL_ = 42.74 mg/g). In both cases, the values of q_mL_ obtained are higher than the highest adsorbed amount measured indicating that saturation was not achieved in the range of concentrations used.

The Freundlich equation, usually used to describe adsorption on heterogeneous surfaces without saturation, showed better fit than Langmuir for MIP-4 (R^2^ = 0.992) and especially for NIP-4 (R^2^ = 0.989). Furthermore, for NIP-4 lower K_F_ and n_F_ values were obtained, than for MIP-4, which probably reflects non-specific adsorption typical of the NIP matrix. In particular, taking into account that the numerical value of K_F_ corresponds to the numerical value of q_e_ for C_e_ = 1 mg/L, the slightly higher value of K_F_ for MIP-4 together with the slightly higher value of q_mL_, indicate that MIP-4 adsorbs slightly more donepezil·HCl than NIP-4. In conclusion, both models indicate that MIP-4 provides more homogeneous and selective adsorption sites for donepezil·HCl, confirming the effectiveness of the molecular imprinting cavities in enhancing adsorption specificity and capacity compared to the non-imprinted polymer.

### 3.6. Morphology (SEM) and Elemental Analysis (SEM-EDX)

**Figure 7:**
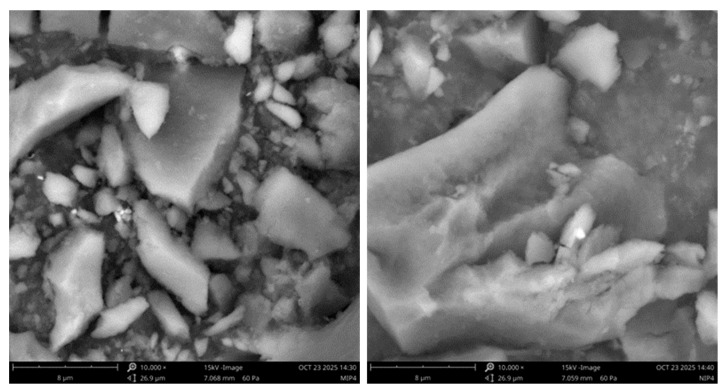
SEM micrographs of MIP-4 (**left**) and NIP-4 (**right**) recorded at 15 kV with a magnification of 10,000×.

Only the MIP-4 and NIP-4 polymers, the most promising ones, were analyzed for their morphology by SEM (Scanning Electron Microscopy). Their micrographs, displayed in Figure 7, reveal irregular particle shapes, which are characteristic of polymers synthesized by bulk polymerization followed by grinding. MIP-4 displayed a more homogeneous morphology, attributed to the structural organization promoted by the presence of the template during polymerization. In contrast, NIP-4 exhibited a more heterogeneous and disordered surface due to the absence of the template, leading to a less organized polymeric network. These morphological differences confirm the successful imprinting process and correlate well with the enhanced binding and controlled release performance observed for MIP-4.

The elemental composition of unloaded and donepezil·HCl-loaded MIP-4 and NIP-4 was evaluated by SEM–EDX analysis, with the corresponding spectra and elemental compositions provided in the Supplementary Materials (Figures S6 and S7 and Table S1). This technique was used to qualitatively assess template removal and drug incorporation through the detection of nitrogen (N) and chlorine (Cl), characteristic elements of donepezil·HCl.

No N or Cl signals were detected in the unloaded polymers, indicating effective template removal after Soxhlet extraction. After drug loading, both MIP-4 and NIP-4 showed the presence of both N and Cl (in the EDX analysis) confirming the incorporation of donepezil·HCl at the polymer surface. These elements were detected at low but consistent levels (1–2 at%), indicating surface-associated loading rather than bulk incorporation.

Overall, SEM–EDX provides qualitative evidence supporting both efficient template removal and subsequent drug loading, complementing the adsorption and release data. These observations are in agreement with FTIR-ATR results (Figure 4), which showed subtle but reproducible spectral changes after drug loading, consistent with interactions between donepezil·HCl and the polymer matrix.

### 3.7. Hydrodynamic Diameter and Zeta Potential

The hydrodynamic diameter, polydispersity index (PDI) and zeta potential of MIP-4 and NIP-4 were determined in water and buffer solutions at different pH values (Table 4). In water, MIP-4 presented smaller and more uniform particles (255.9 ± 8.6 nm, PDI = 0.428) compared to NIP-4 (466.3 ± 12.2 nm, PDI = 0.916), both showing strongly negative zeta potentials (−29.0 ± 0.75 and −28.1 ± 1.10 mV), attributed to the deprotonation of the carboxylic acid groups from acrylic acid (pKa ≈ 4.5 [46]), which exist predominantly in the –COO^−^ form at neutral pH. However, the high PDI value observed for NIP-4 indicates particle aggregation, as PDI values greater than 0.8 are generally associated with aggregated systems. MIP-4 and NIP-4 were also analysed at pH 3.0, both polymers showed increased particle sizes (2441 ± 587 nm for MIP-4 and 892.2 ± 209 nm for NIP-4) and less negative zeta potentials (−2.57 ± 0.27 and −3.32 ± 1.04 mV), suggesting aggregation due to protonation of carboxylic groups of the polymer. At pH 5.5, particle sizes decreased (1408 ± 192 nm for MIP-4 and 439.4 ± 29.2 nm for NIP-4) with moderately negative zeta potentials (−13.4 ± 0.46 and −10.3 ± 0.47 mV), likely due to charge compensation between protonated and deprotonated –COOH/–COO^−^ groups near neutral conditions. At pH 7.0, both polymers exhibited a distinct size increase (4843 ± 109 nm for MIP-4 and 978.9 ± 109 nm for NIP-4) and more negative zeta potentials (−16.9 ± 0.42 and −12.7 ± 0.75 mV), due to the presence of ionized carboxylate (–COO^−^) groups. This indicates enhanced electrostatic repulsion between ionized carboxylate groups, leading to partial expansion or loosening of the polymer network.

Generally, the results confirm that both surface charge and particle size are strongly pH-dependent and differ between MIP-4 and NIP-4, which may influence the subsequent drug release behaviour. In addition, the PDI values of NIP-4 in water and at pH3 and 7 are higher, indicating particle aggregation, which could also affect the release profile of NIP-4.

### 3.8. Evaluation of the Effect of MIPs and NIPs on Cellular Viability

**Figure 8:**
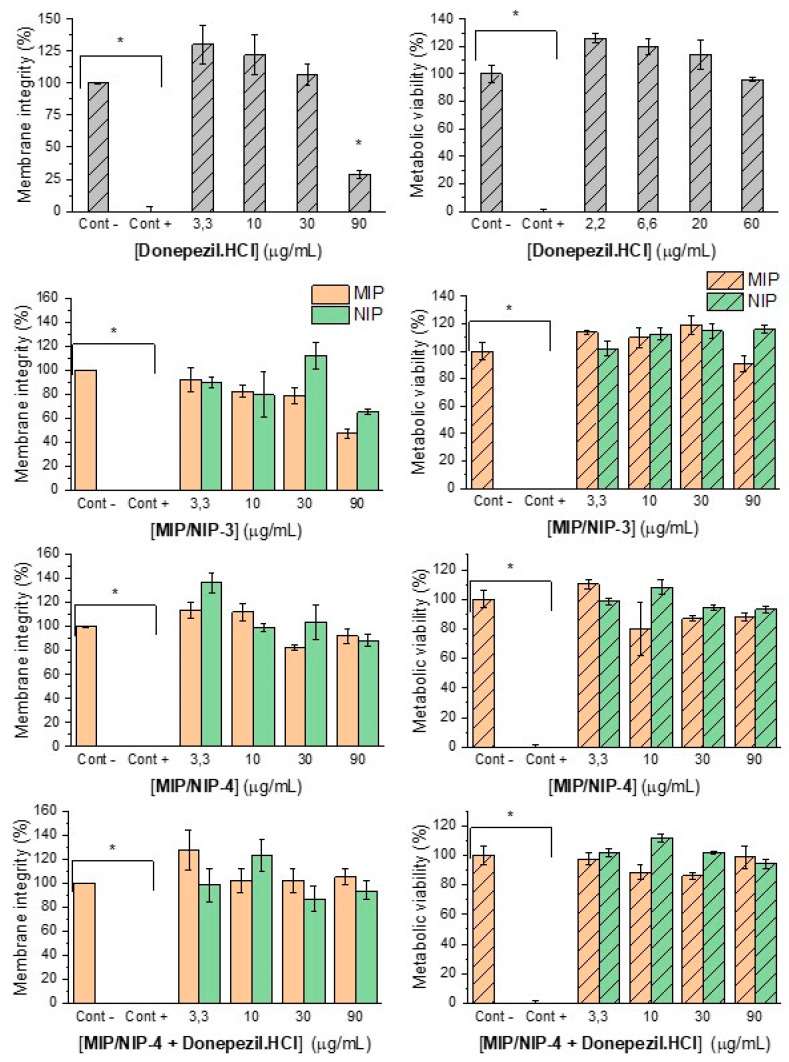
Study of the effect of MIP/NIPs on the cell viability of MCF-7 cells. (**Left**) represents membrane integrity assessed by FDA assay and (**right**) represents metabolic viability assessed by WST-8 assays (striped bars). Results are expressed as percentage of viability relative to the negative control (no stimulus) and the positive control (SDS treatment), and are presented as mean ± SEM (n ≥ 4). Statistical analysis was performed using the Tukey–Kramer multiple comparisons test, with significant differences (*p* > 0.05) indicated relative to the negative control (*).

Cell viability assays are crucial to evaluate the cytotoxicity of the prepared polymers. Only the most promised were tested, namely MIP/NIP-3 and -4, and also the MIP-4 loaded with donepezil·HCl. These polymers were tested at a range of concentrations of 3.3, 10, 30, and 90 µg/mL using the human breast cancer cell line MCF-7 as a human cellular model. Two assays were used to access cellular viability, fluorescein diacetate (FDA) and WST-8, for membrane integrity and metabolic viability assessment, respectively. FDA can passively diffuse across the cell membrane due to its nonpolar nature. Once inside the cell, FDA is hydrolyzed by intracellular esterases generating the polar compound fluorescein, which is trapped inside cells with intact membranes. However, fluorescein leaks out when the membrane integrity is compromised. Therefore, cells with intact membranes display higher intracellular fluorescence levels compared to cells with reduced viability or membrane damage. As shown in Figure 8-left panel, the tested compounds did not negatively affect membrane permeability. It was also observed that donepezil had an adverse effect on membrane integrity at 90 µg/mL, a concentration that was not evaluated in the WST-8 assay. The WST-8 assay was employed to evaluate the metabolic viability of the MCF-7 cell line. This method is based on the ability of metabolically active cells to reduce the tetrazolium salt WST-8 into formazan, which can be quantified spectrophotometrically. As shown in Figure 8 (right panel) none of the tested polymers induced a significant loss of cell viability within the evaluated concentration range (3.3–90 µg/mL). The effect of donepezil was also evaluated at concentrations of 2.2 and 60 µg/mL, showing no interference with metabolic processes. This observation is consistent with its well-established safety profile, supporting its long-term clinical use in the treatment of Alzheimer’s disease.

Overall, these results indicate a high level of safety for the synthesized polymers at the tested concentrations and within the cellular model employed in this study, supporting their potential suitability for further biological evaluation. Further investigations on additional cell lines, principally non-tumorigenic and neuronal cells, will be important to gain a more comprehensive understanding of the cytotoxic potential and biocompatibility of these materials.

### 3.9. Effect of pH on the In Vitro Release of Donepezil

**Figure 9:**
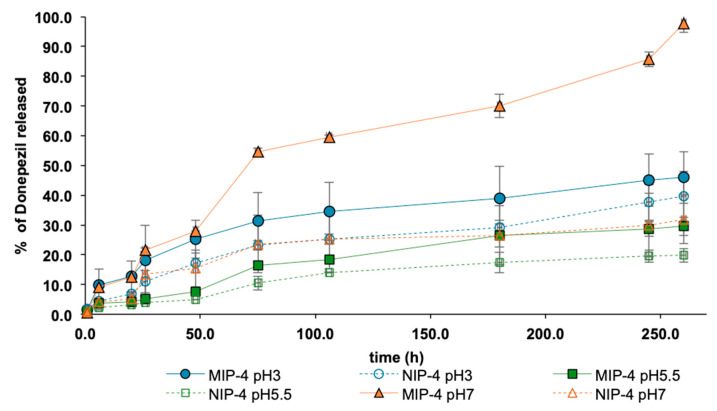
Release curves of donepezil·HCl from MIP-4 and NIP-4 under pH 3, 5.5 and 7.

For the in vitro release studies, the MIP-4 and its corresponding NIP-4 were selected, based on their performance in previous retention and by revealing no cytotoxicity. The polymers MIP-4 and NIP-4 were loaded with donepezil·HCl. The release assays were conducted in three physiologically relevant buffer systems with pH values of 3, 5.5, and 7 to evaluate the pH-dependent release profile of donepezil·HCl. Figure 9 shows the release curves of donepezil·HCl in the three different pH media.

After 11 days, the lowest cumulative release was observed at pH 5.5 (~30%), followed by pH 3.0 (~46%) and pH 7.0, where the maximum release reached 98%. Surprisingly, the release of the drug from NIP-4 was consistently lower than that of the MIP at all pH values and, furthermore, did not follow a similar trend (pH 3.0, ~40%; pH 5.5, ~20%; pH 7.0, ~32%), even though the NIP at pH 7.0 would be expected to show the highest cumulative release value. The lower cumulative release of the drug from the NIP compared to the MIP was unexpected, as NIPs usually release more due to lack of specific binding sites [47]. This behavior may be associated with the combined use of tetramethylquercetin-based monomer **1** and acrylic acid as a pH-sensitive monomer, representing a novel approach in molecular imprinting. In the NIP, non-specific interactions, including π–π stacking, dipole–dipole and hydrophobic interactions arising from the tetramethylquercetin units in the polymer, may contribute to partial retention of donepezil, with electrostatic interactions playing a minor role. In the MIP, template-directed polymerization organizes flavonoid and acrylic acid units within imprinted cavities, allowing cooperative stabilization through π–π, dipole–dipole and hydrophobic interactions, and hydrogen bonding, resulting in a more controlled, pH-dependent release profile. Overall, the observed release behavior reflects the interplay between these non-covalent interactions and the pH-responsive properties of the polymer, highlighting the distinctive behavior of this molecular imprinting system. These differences could be explained by variations in zeta potential and hydrodynamic size between the MIP and NIP at the same pH (Table 4). Changes in surface charge and particle size affect drug–polymer interactions and diffusion, contributing to the superior and pH-responsive release observed for the MIP.

To elucidate the release mechanism of donepezil·HCl from MIP-4 and NIP-4, the Korsmeyer–Peppas model was applied. This model describes drug release from controlled delivery systems by correlating the fraction of drug released with time. According to this model, a diffusion exponent (*n*) ≤ 0.5 indicates Fickian diffusion, *n* = 1 corresponds to zero-order release, and values between 0.5 and 1 suggest anomalous (non-Fickian) transport, involving both diffusion and polymer relaxation mechanisms (Table 5) [37].

At pH 3, the Korsmeyer–Peppas parameter *n* for both MIP-4 (0.51) and NIP-4 (0.52) is close to 0.5, indicating a predominantly Fickian diffusion release mechanism. This aligns with the protonated state of the –COOH groups in the polymer and donepezil·HCl (pKa = 8.9 [35]), favoring hydrogen bonding and π–π, dipole–dipole and hydrophobic interactions in the imprinted cavities that facilitate diffusion-controlled release. The R^2^ values at this pH are reasonably high (0.90 for MIP-4 and 0.97 for NIP-4), supporting the model fit. The zeta potential values near neutral (−2.57 mV for MIP-4 and −3.32 mV for NIP-4) suggest minimal electrostatic repulsion (Table 4).

At pH 5.5, *n* increases to 0.62 (MIP-4) and 0.65 (NIP-4), suggesting anomalous (non-Fickian) transport involving both diffusion and polymer relaxation mechanisms. The corresponding R^2^ values (0.91 and 0.89) indicate a good fit. The more negative zeta potentials (−13.4 mV and −10.3 mV) imply stronger electrostatic contributions and non-covalent interactions between the drug and polymer, without neglecting the contribution of hydrogen bonding, potentially affecting the release behaviour (Table 4). The significant increase in average of hydrodynamic diameter for MIP-4 and PDI may reflect changes in the polymer matrix, although swelling was not directly measured.

At pH 7, the *n* value for MIP-4 increases to 0.70, indicating non-Fickian release controlled by a combination of diffusion and polymer relaxation mechanisms. The R^2^ value (0.93) confirms a good model fit. At this pH, the carboxylic acid groups of the polymer (pKa ≈ 4.5) are mainly ionized, resulting in increased negative charges along the polymer chains, as supported by the zeta potential value of −16.9 mV. This electrostatic repulsion between negatively charged polymer chains could lead to expansion or loosening of the polymer network, facilitating greater drug diffusion and release, also evidenced by the increase in the hydrodynamic diameter of the particles (Table 4). In contrast, NIP-4 shows an *n* value near 0.52 (R^2^ = 0.94), indicating Fickian diffusion-dominated release, consistent with its smaller particle size and less negative zeta potential (−12.7 mV), reflecting weaker electrostatic effects and less structural rearrangement.

In summary, the release of donepezil·HCl from MIP-4 is pH-dependent and governed by diffusion combined with polymer matrix interactions and potential structural changes, as supported by the variation in n, R^2^, zeta potential, and particle size. For NIP-4, drug release is principally diffusion-controlled with less pronounced pH effects, likely due to the absence of specific binding sites, as explained before.

In conclusion, MIP-4 demonstrated pH-sensitive behaviour, releasing donepezil·HCl efficiently under physiological conditions (pH 7.4), which simulates blood plasma. This profile supports the use of MIP-4 as a functional matrix in controlled drug delivery systems, with potential to enhance the bioavailability and targeted delivery of donepezil·HCl.

### 3.10. Evaluation of Cholinesterase Inhibition

One of the objectives of this work was to study whether quercetin-based monomer **1** and the resulting polymers exhibit intrinsic anticholinesterase activity. Therefore, monomer **1** and selected polymers, including MIP-4 and NIP-4, and polymer loaded with donepezil·HCl were evaluated as potential inhibitors of acetylcholinesterase (AChE) and butyrylcholinesterase (BuChE) using the Ellman method, Table 6. As a reference, donepezil·HCl, a clinically approved cholinesterase inhibitor and the target drug for delivery, was used under previously optimized assay conditions [48], across a concentration range of 0.3–312 nM. Dose–response curves were generated (enzyme inhibition *vs.* log [inhibitor]), allowing for accurate determination of the percentage of inhibition. The calculated IC_50_ values for donepezil·HCl were 60.97 ± 0.62 nM for *ee*AChE and 3.09 ± 0.39 µM for eqBuChE, consistent with literature data and confirming the reliability of the assay.

As expected, donepezil·HCl at 100 µM inhibited both enzymes by 100%, validating its reference role. Monomer **1**, tested at 200 µM, exhibited low inhibitory activity: only 15.5% inhibition for *ee*AChE and 25.1% for eqBuChE, suggesting some selective interaction with eqBuChE. MIP-4 and NIP-4 without loaded drug, also showed weak inhibition toward *ee*AChE (13.5% and 5.1%, respectively) and no effect against eqBuChE at 30.0 µg/mL, indicating those polymers have insignificant intrinsic activity, which is desirable for controlled delivery systems. Notably, polymers loaded with donepezil·HCl (0.7 µg for MIP-4 and 0.6 µg for NIP-4) demonstrated significant inhibition of *ee*AChE (~52%), confirming successful encapsulation and subsequent drug release at biologically active concentrations, which reinforces the potential of MIPs as drug delivery platforms.

The lack of inhibition against eqBuChE in these formulations may be attributed to the insufficient released drug levels, which are likely insufficient to reach the IC_50_ required for this enzyme.

Overall, MIP-4 stands out as a promising dual-function material: capable of drug encapsulation and release, and with mild intrinsic bioactivity that could contribute synergistically in therapeutic contexts.

### 3.11. Antioxidant Capacity by the Ferric Reducing Antioxidant Power (FRAP) Assay

**Figure 10:**
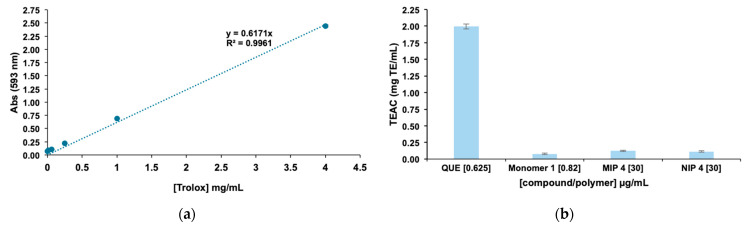
FRAP assay results. (**a**) Calibration curve obtained with Trolox (y = 0.6171x; R^2^ = 0.9961) at 593 nm. (**b**) Antioxidant capacity of quercetin (QUER, 0.625 µg/mL), monomer **1** (0.021 µg/mL), MIP-4, and NIP-4 expressed as Trolox equivalent antioxidant capacity (TEAC, mg TE/mL). Data are presented as mean ± SD (n = 3).

The antioxidant activity of the QUER, monomer **1**, MIP-4 and NIP-4 was evaluated by the FRAP assay, which measures the capacity of samples to reduce the Fe^3+^–TPTZ complex to Fe^2+^–TPTZ at 593 nm. The Trolox calibration curve (y = 0.6171x; R^2^ = 0.9961) was used to calculate the TEAC values. Quercetin displayed high reducing power (≈2.0 mg TE/mL), while the monomer **1**, MIP-4, and NIP-4 presented very low activity (Figure 10). These results are consistent with the structural modification of quercetin, as its hydroxyl groups, mainly responsible for its antioxidant activity, are protected in monomer **1**. Nevertheless, the lack of antioxidant activity also highlights the excellent chemical stability and robustness of the polymer network, which resists oxidative degradation and maintains its structural integrity throughout the delivery process. This stability is advantageous for the development of reliable, long-circulating drug delivery systems, particularly in biological environments where oxidative stress is prevalent.

## 4. Conclusions

In conclusion, this work introduces a novel molecularly imprinted polymer based on a quercetin-derived functional biomonomer (monomer **1**), representing a proof of concept for the use of natural product-derived monomers in MIP-based systems for donepezil·HCl loading and release. The optimized formulation, MIP-4, prepared from a 1:1 mixture of monomer **1** and acrylic acid, exhibited pronounced pH-responsive behavior and a controlled release profile under physiologically relevant conditions compared to its non-imprinted counterpart NIP-4, reflecting the contribution of imprinting-induced interactions, despite the moderate imprinting effect.

The intrinsic fluorescence of the quercetin-derived monomer, combined with low cytotoxicity and effective donepezil·HCl release leading to *ee*AChE inhibition, highlights the multifunctional character of the material. These results should be interpreted cautiously, within the limitations of the model systems employed.

Overall, this materials-oriented study demonstrates the feasibility of using natural product-derived monomers for the construction of molecularly imprinted polymers with potential applications in controlled drug delivery, clearly framed as a proof-of-concept.

## Figures and Tables

**Table 1:** Synthesized molecularly imprinted polymers, functional monomers and crosslinking agents used, and their respective quantities.

MIP/NIP	Monomer 1 (eq.; mmol; mg)	Acrylic Acid (eq.; mmol; µL)	Crosslinker (eq.; mmol; mL)
MIP/NIP-1	(1; 0.0791; 32.6)	(3; 0.237; 16.3)	EGDMA (14; 1.1; 0.21)
MIP/NIP-2	(2; 0.158; 65.2)	(2; 0.158; 11)	EGDMA (14; 1.1; 0.21)
MIP/NIP-3	(1; 0.0791; 32.6)	(3; 0.237; 16.3)	TMPTA (14; 1.1; 0.35)
MIP/NIP-4	(2; 0.158; 65.2)	(2; 0.158; 11)	TMPTA (14; 1.1; 0.35)

**Table 2:** Adsorption capacity (qₑ) for an initial concentration of 90.0 mg/L and retention rate (RR, %) of the synthesized MIPs and NIPs.

Polymer	q_e_ (mg/g)	RR (%)	Polymer	q_e_ (mg/g)	RR (%)
MIP-1	26.48 ± 0.67	29.42 ± 0.80	NIP-1	25.85 ± 1.54	28.72 ± 1.71
MIP-2	17.24 ± 0.80	19.15 ± 0.89	NIP-2	17.76 ± 0.45	19.73 ± 0.50
MIP-3	34.54 ± 1.40 *	38.37 ± 1.56	NIP-3	30.84 ± 1.38	34.27 ± 1.53
MIP-4	32.90 ± 0.50 **	36.56 ± 0.56	NIP-4	27.50 ± 0.40	30.56 ± 0.44

Data are presented as mean ± SD of three independent replicates. Statistical analysis was performed using ANOVA. * indicates significant differences (*p* < 0.05), and ** indicates highly significant differences (*p* < 0.01), between MIP and the corresponding NIP.

**Table 3:** Results from the application of Langmuir and Freundlich equations to the adsorption isotherms of donepezil·HCl on MIP-4 and NIP-4.

	Langmuir			Freundlich		
Polymer	q_mL_ (mg/g)	K_L_ (L/mg)	R^2^	K_F_ ((mg/g)/(mg/L)^1/nF^)	n_F_	R^2^
MIP-4	43.86	0.049	0.975	5.70	2.4	0.992
NIP-4	42.74	0.039	0.929	4.87	2.3	0.989

**Table 4:** Hydrodynamic diameter, polydispersity index (PDI) and Zeta potential of MIP-4 and NIP-4 measured in different dispersant media: ultrapure water, trisodium citrate buffer (pH 3.0), and phosphate buffer (pH 5.5 and pH 7.0). Data are presented as mean ± standard deviation (n = 3).

Dispersant	Polymer	Average Size (nm)	PDI	Zeta Potential (mV)
H_2_O	MIP-4 NIP-4	255.9 ± 8.6 466.3 ± 12.2	0.428 0.916	−29.0 ± 0.7 −28.1 ± 1.1
Trisodium Citrate buffer pH3	MIP-4 NIP-4	2441 ± 587.0 892.2 ± 209.0	0.314 0.859	−2.57 ± 0.3 −3.32 ± 1.0
Phosphate buffer pH5.5	MIP-4 NIP-4	1408.0 ± 192.2 439.4 ± 29.2	0.868 0.700	−13.4 ± 0.5 −10.3 ± 0.5
Phosphate buffer pH7	MIP-4 NIP-4	4843.0 ± 109.2 978.9 ± 109.2	0.113 0.739	−16.9 ± 0.4 −12.7 ± 0.8

**Table 5:** Korsmeyer-Peppas model parameters for donepezil·HCl release from MIP-4 and NIP-4 at pH 3, 5.5, and 7.

Parameters	pH3		pH5.5		pH7	
	MIP-4	NIP-4	MIP-4	NIP-4	MIP-4	NIP-4
*n*	0.51 ± 0.003	0.52 ± 0.032	0.62 ± 0.102	0.65 ± 0.229	0.70 ± 0.103	0.52 ± 0.022
*k*	0.062 ± 0.001	0.05 ± 0.005	0.03 ± 0.016	0.03 ± 0.030	0.02 ± 0.013	0.06 ± 0.002
*R* ^2^	0.90 ± 0.108	0.97 ± 0.001	0.91 ± 0.037	0.89 ± 0.094	0.93 ± 0.055	0.94 ± 0.043

**Table 6:** Inhibitory effects of donepezil·HCl, monomer **1**, and MIP/NIP-4 in both unloaded and loaded forms.

Compounds/Polymers	Concentration	Inhibition (%)	
		*ee*AChE	eqBuChE
Donepezil·HCl	100.0 µM	100.0 ± 0.6	100.0 ± 5.8
Monomer **1**	200.0 µM	15.5 ± 3.2	25.1 ± 5.8
MIP-4	30.0 µg/mL	13.5 ± 1.5	NI
NIP-4	30.0 µg/mL	5.1 ± 3.3	NI
MIP-4+ Donepezil·HCl (0.7 µg)	30.0 µg/mL	52.2 ± 0.7	NI
NIP-4+ Donepezil·HCl (0.6 µg)	30.0 µg/mL	51.7 ± 1.8	NI

NI: No Inhibition.

## Data Availability

The original contributions presented in this study are included in the article/Supplementary Material. Further inquiries can be directed to the corresponding author.

## Supplementary Materials

The following supporting information can be downloaded at: https://www.mdpi.com/article/10.3390/polym18020234/s1, Figure S1: ^1^H NMR spectrum (400 MHz, CDCl_3_) of 3-hydroxy-3′,4′,5,7-tetramethylquercetin **2**; Figure S2: ^13^C APT NMR spectrum (100 MHz, CDCl_3_) of 3-hydroxy-3′,4′,5,7-tetramethylquercetin **2**; Figure S3: ^1^H NMR spectrum (400 MHz, CDCl_3_) of 3-acryloxy-3′,4′,5,7-tetramethylquercetin **1**; Figure S4: ^13^C APT NMR spectrum (100 MHz, CDCl_3_) of 3-acryloxy-3′,4′,5,7-tetramethylquercetin **1**; Figure S5. (A) UV–Vis spectra of donepezil·HCl in water at different concentrations; the inset shows the calibration curve obtained by plotting absorbance at 270 nm vs. donepezil·HCl concentration. (B) UV–Vis spectra of the extracts obtained after the polymer extraction procedure, used to assess the removal of the template (donepezil·HCl); Figure S6: SEM images showing the surface morphology of MIP-4 and NIP-4 before and after loading with donepezil·HCl. (recorded at 15 kV with a magnification of 1000×); Figure S7: EDX spectra showing the elemental composition of MIP-4 and NIP-4 before and after loading with donepezil·HCl; Table S1. Surface elemental composition of MIP-4 and NIP-4 before and after loading with donepezil·HCl, determined by SEM–EDX analysis. Results are expressed as mean ± standard deviation (n = 12 analysis points).
