# Supplementary material for: Novel Bioinspired Quercetin-Based Polymers for the Sustained Release of Donepezil in Alzheimer’s Disease Therapy
Source: Polymers (Basel). 2026 Jan 16;18(2):234. doi: 10.3390/polym18020234 (PMC12845978; doi:10.3390/polym18020234)
Supplement: Supplementary file 1 [file polymers-18-00234-s001.zip › polymers-4015047-supplementary.pdf]

Supplementary Materials

Novel Bioinspired Quercetin-Based Polymers for the Sustained Release of Donepezil in Alzheimer's Disease Therapy

**Elisabete P. Carreiro [1,*], Pedro Múria [2], Diogo Velez [2], Manuela R. Carrott [1,2], Anthony J. Burke [3,4,5]
and Ana R. Costa [6,7]**

[1] Institute for Research and Advanced Training (IIFA), LAQV-REQUIMTE, University of Évora, Rua Romão Ramalho, 59, 7000-671 Évora, Portugal; manrc@uevora.pt
[2] Department of Chemistry and Biochemistry, School of Science and Technology, University of Évora, 7000-671 Évora, Portugal; pmuria@unilicungo.ac.mz (P.M.); diogofrazao31@gmail.com (D.V.)
[3] Coimbra Chemistry Centre, Institute of Molecular Sciences (CQC-IMS), Departamento de Química, University of Coimbra, 3004-535 Coimbra, Portugal; ajburke@ff.uc.pt
[4] Pharmaceutical Chemistry Laboratory, Faculty of Pharmacy, Pólo das Ciências da Saúde, University of Coimbra, Azinhaga de Santa Comba, 3000-548 Coimbra, Portugal
[5] CNC-UC-Center for Neuroscience and Cell Biology, CIBB-Center for Innovative Biomedicine and Biotechnology, University Coimbra, 3004-504 Coimbra, Portugal
[6] Department of Medical & Health Sciences, School of Health & Human Development, University of Évora, 7000-810 Évora, Portugal; acrc@uevora.pt
[7] IIFA, CREATE-Center for Sci-Tech Research in Earth System and Energy, University of Évora, 7000-810 Évora, Portugal
* Correspondence: betepc@uevora.pt

NMR Spectrum of Compound 3-Hydroxy-3′,4′,5,7-Tetramethylquercetin 2

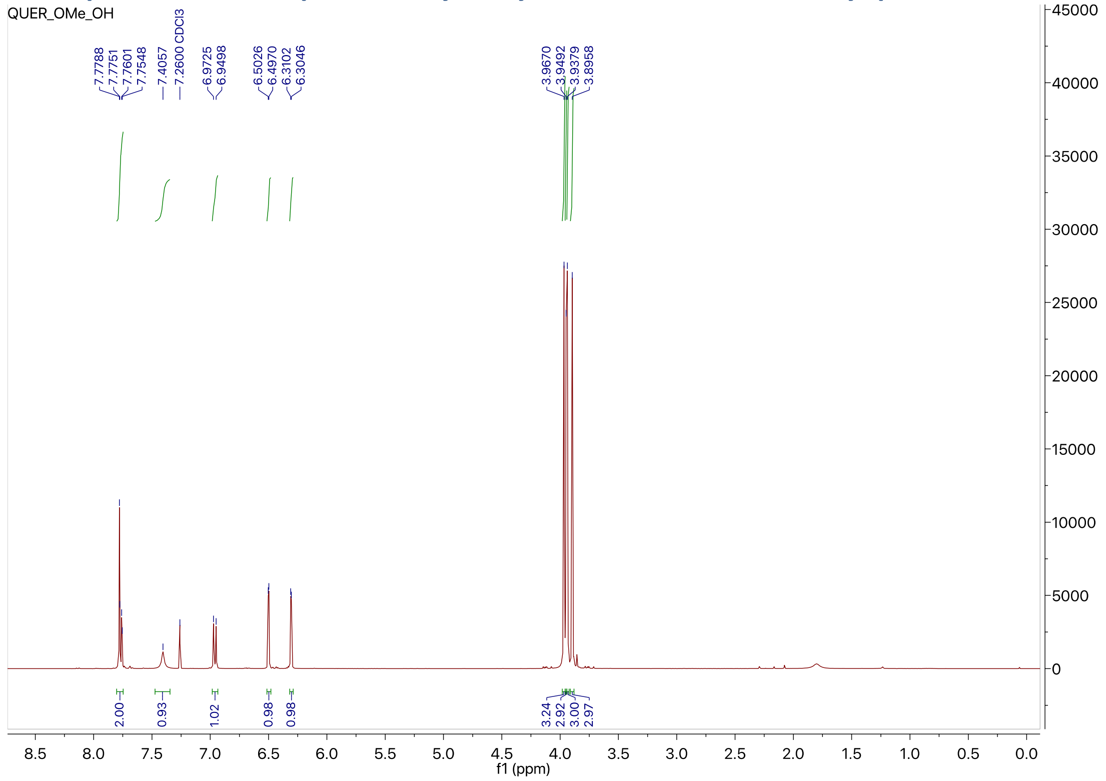

Figure S1. ^1H NMR spectrum (400 MHz, $CDCl_3$) of 3-hydroxy-3′,4′,5,7-tetramethylquercetin **2**.

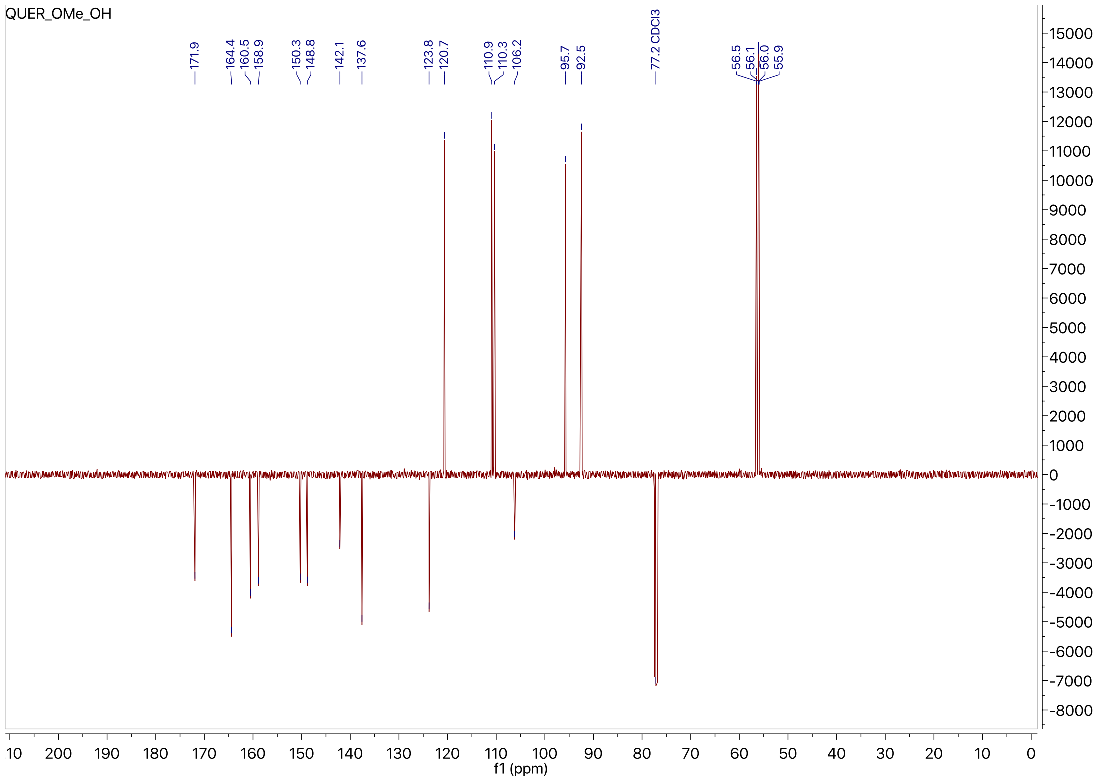

Figure S2. ^{13}C NMR spectrum (100 MHz, $CDCl_3$) of 3-hydroxy-3′,4′,5,7-tetramethylquercetin **2**.

NMR Spectrum of Compound 3-acryloxy-3’,4’,5,7-tetramethylquercetin 1

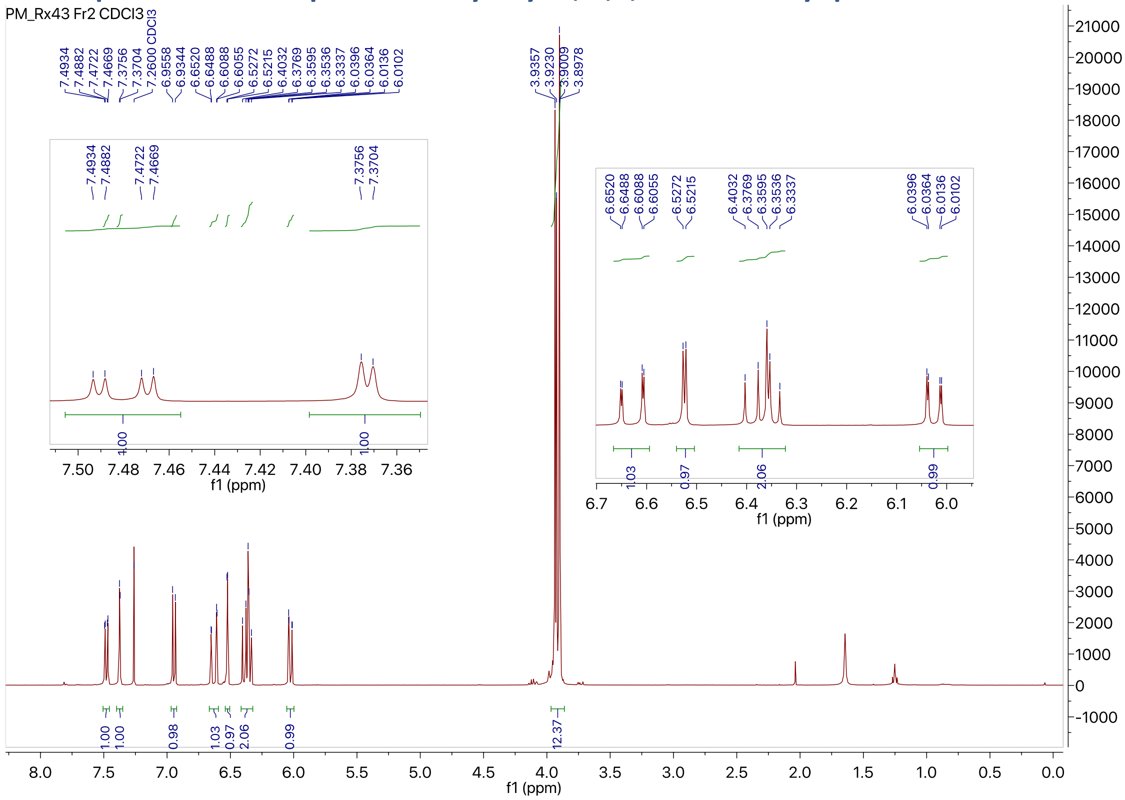

Figure S3. ¹H NMR spectrum (400 MHz, $CDCl_3$) of 3-acryloxy-3’,4’,5,7-tetramethylquercetin **1**.

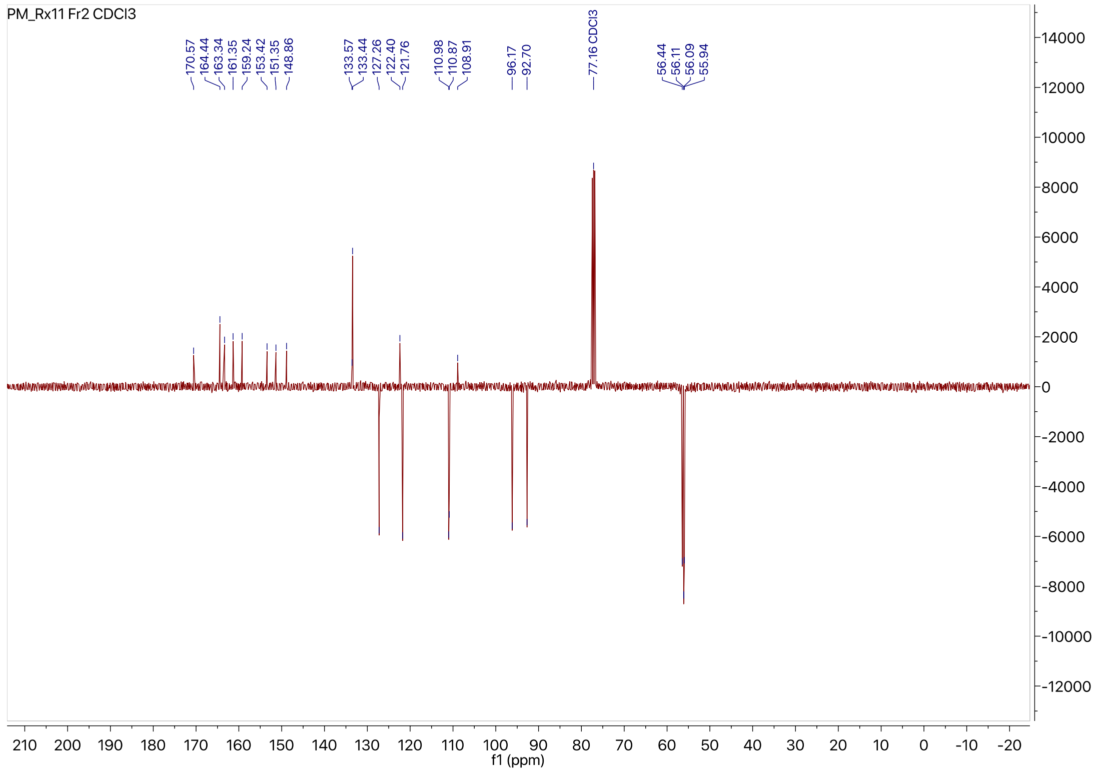

Figure S4. ^{13}C NMR spectrum (100 MHz, $CDCl_3$) of 3-acryloxy-3',4',5,7-tetramethylquercetin **1**.

Experimental Procedure for Confirmation of Complete Removal of Donepezil·HCl from the MIP-4 Matrix after Soxhlet Extraction

To confirm the efficient removal of donepezil·HCl from the polymer matrix after Soxhlet extraction, 26.01 mg of MIP-4, 28.99 mg of NIP-4, and 17.29 mg of donepezil·HCl-loaded MIP-4 (containing 364.68 µg of encapsulated donepezil·HCl) were each placed into separate 15 mL Falcon tubes. Subsequently, 5 mL of a methanol/acetic acid mixture (MeOH:AcOH, 6:4 v/v) was added to each tube, and the samples were subjected to ultrasonic treatment for 1 h. After sonication, the samples were centrifuged at 4500 rpm for 5 min, and the supernatants were collected. The polymers were then re-extracted with an additional 5 mL of the same solvent mixture and sonicated for 30 min, followed by centrifugation and collection of the supernatants. This procedure was followed by three additional washing cycles using 5 mL of MeOH:AcOH (6:4, v/v), each with 15 min of ultrasonic treatment. All collected supernatants (Ex 1-5) were combined and evaporated under reduced pressure. The resulting residue was redissolved in 1 mL of deionized water. A sixth extraction cycle (Ex 6) was subsequently performed using 500 µL of deionized water under gentle rotation at room temperature for 16 h. After centrifugation, the supernatants were collected and analyzed separately to confirm the absence of donepezil·HCl.
Quantitative analysis was carried out using UV–Vis spectroscopy at 270 nm. A calibration curve for donepezil·HCl in water was constructed over the concentration range of 6.25–300 µg/mL (6.25, 12.5, 25, 50, 100, 200, and 300 µg/mL), yielding a linear regression described by the equation $y = 0.010x + 0.070$ with a correlation coefficient of $R^2 = 0.999$.

Analysis of the collected extracts (Ex 1–5 and Ex 6) confirmed that neither MIP-4 nor NIP-4 contained detectable amounts of donepezil·HCl after soxhlet extration, as no characteristic absorption bands were observed in either the combined organic extracts (Ex 1–5) or the final aqueous extract (Ex 6). In contrast, for the donepezil·HCl-loaded MIP-4 sample, characteristic absorption bands of donepezil·HCl were clearly detected in the combined organic extracts (Ex 1–5), confirming the successful extraction of the drug from the polymeric matrix. In the final aqueous extract (Ex 6), no absorption bands corresponding to donepezil·HCl were observed, confirming its complete removal after the extraction procedure. Representative UV–Vis spectra supporting these findings are shown in Figure S5.

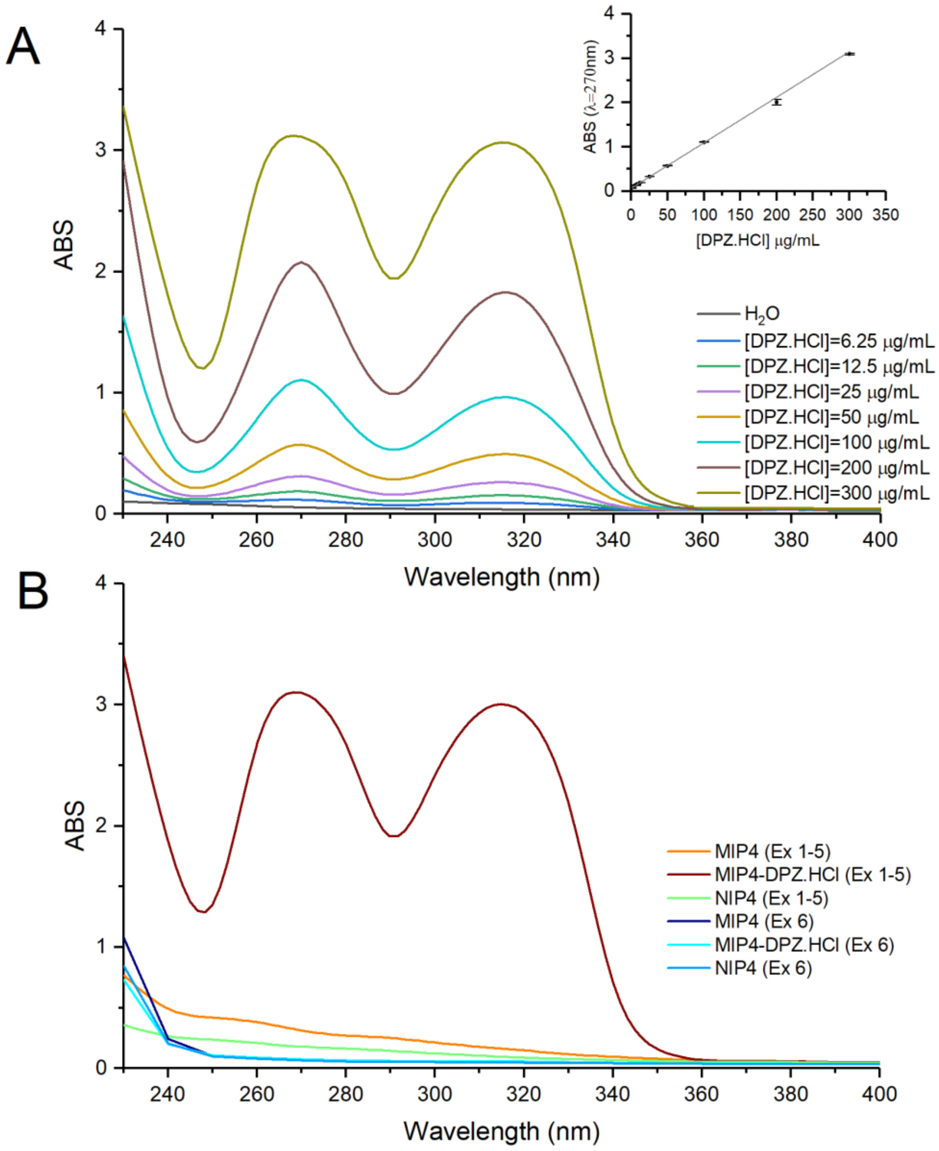

Figure S5. (A) UV–Vis spectra of donepezil·HCl (DPZ.HCl) in water at different concentrations; the inset shows the calibration curve obtained by plotting absorbance at 270 nm *vs* donepezil·HCl concentration. (B) UV–Vis spectra of the extracts obtained after the polymer extraction procedure, used to assess the removal of the template (donepezil·HCl).

SEM – EDX Analysis:

MIP-4

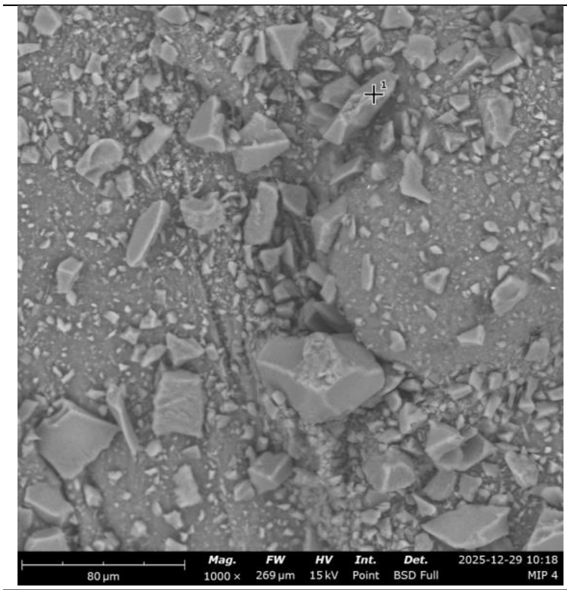

MIP-4 loaded with donepezil.HCl

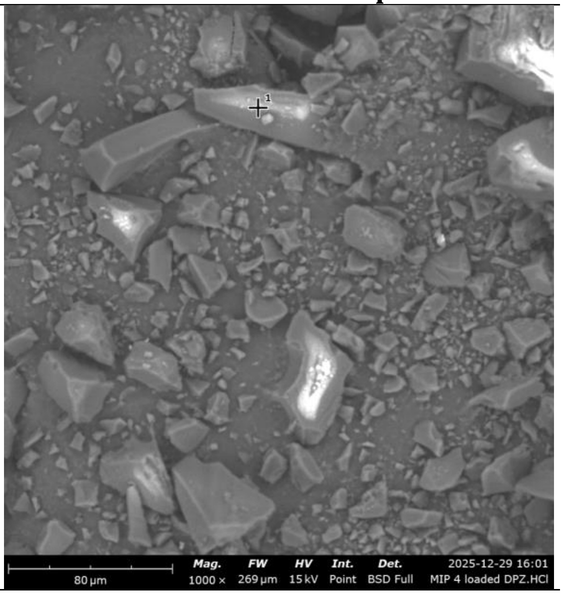

NIP-4

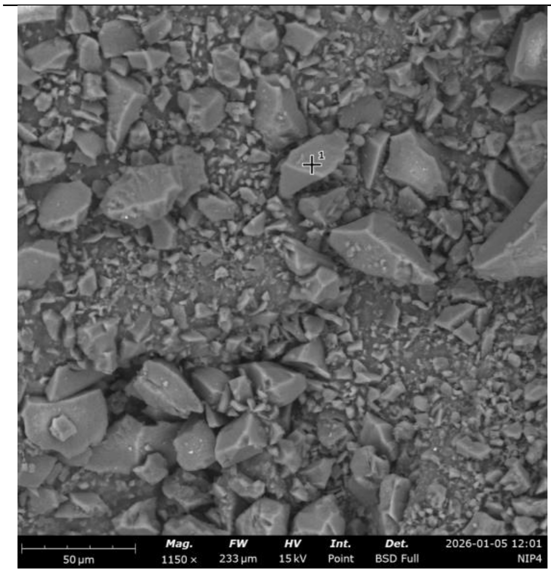

NIP-4 loaded with donepezil.HCl

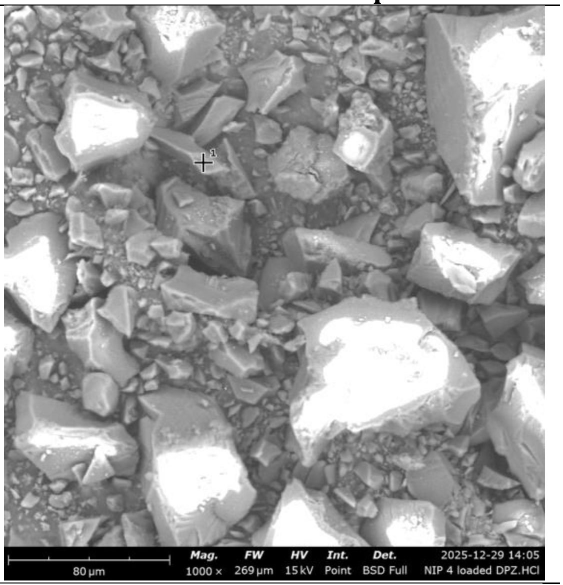

Figure S6. SEM images showing the surface morphology of MIP-4 and NIP-4 before and after loading with donepezil·HCl. (recorded at 15 kV with a magnification of 1,000×)

MIP-4

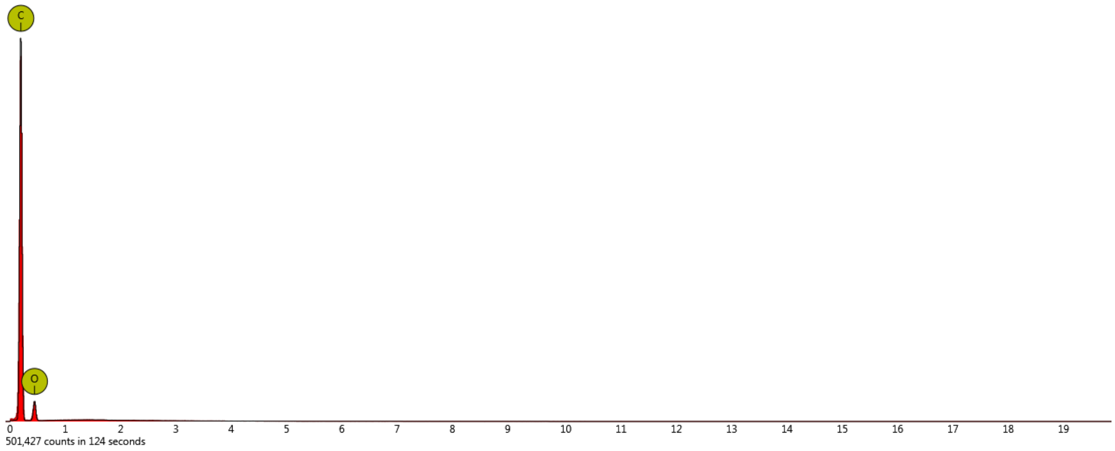

MIP-4 loaded with donepezil.HCl

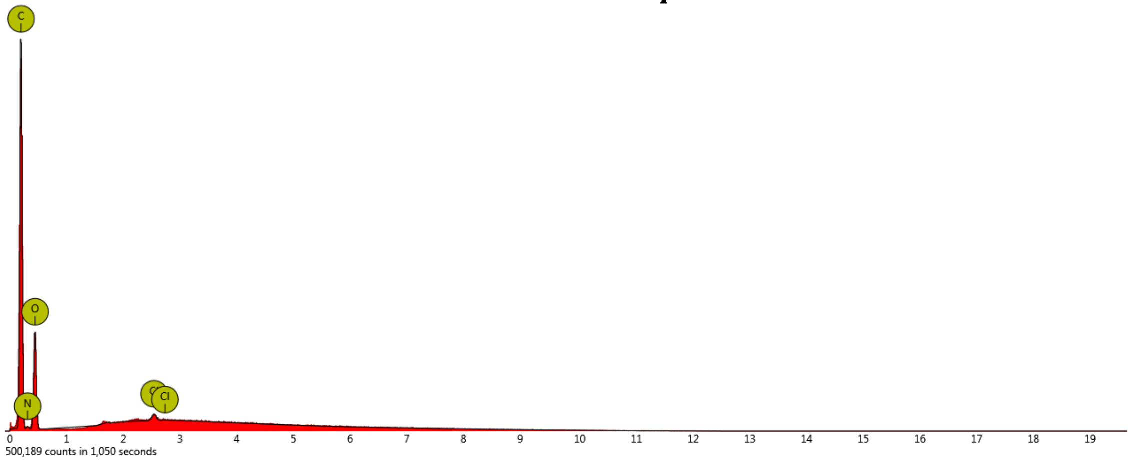

NIP-4

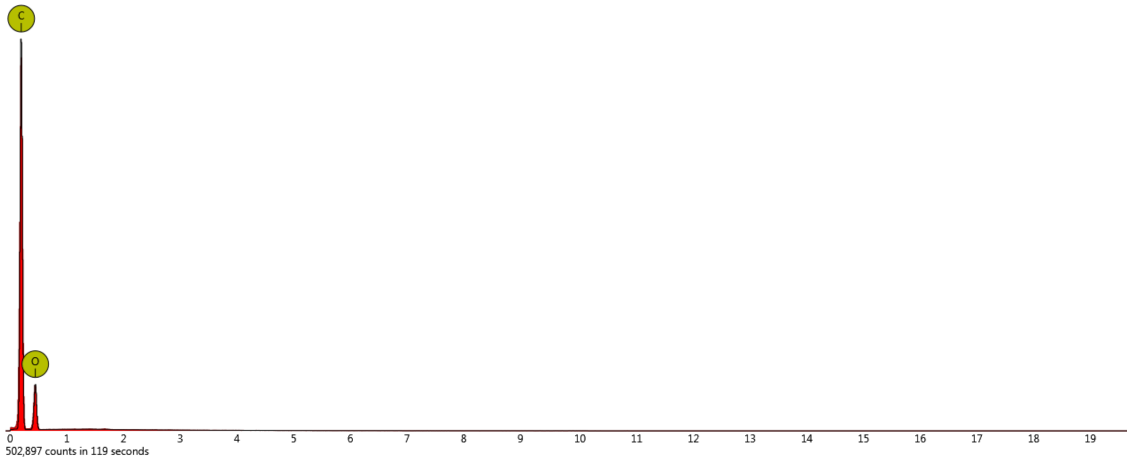

NIP-4 loaded with donepezil.HCl

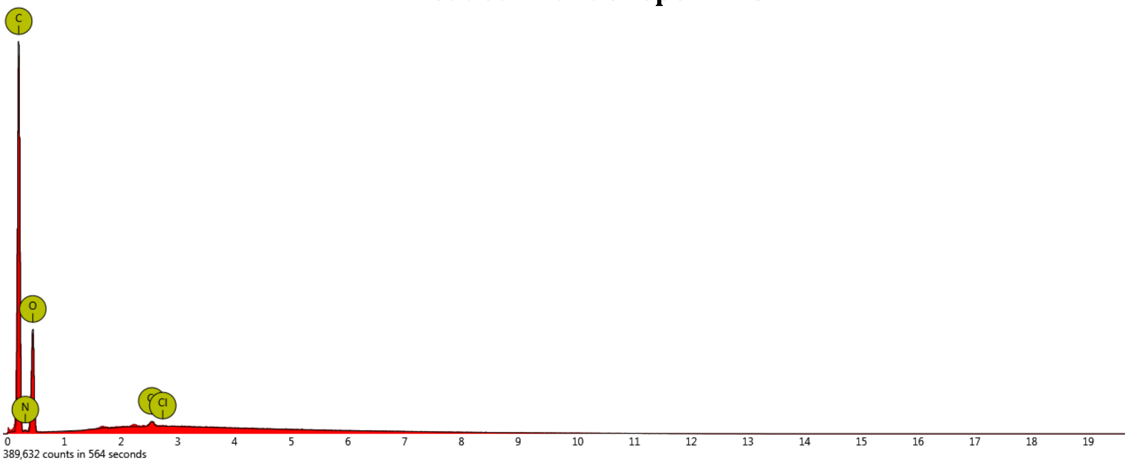

Figure S7. EDX spectra showing the elemental composition of MIP-4 and NIP-4 before and after loading with donepezil·HCl.

Table S1. Surface elemental composition of MIP-4 and NIP-4 before and after loading with donepezil·HCl, determined by SEM–EDX analysis. Results are expressed as mean ± standard deviation (n = 12 analysis points).

	**Oxygen**	**Carbon**	**Nitrogen**	**Chlorine**
	**Atomic (%)**	**Atomic (%)**	**Atomic (%)**	**Atomic (%)**
**MIP-4**	49.9±11.1	50.1±11.1	-	-
**MIP-4 loaded donepezil.HCl**	52.57±9.22	44.88±8.97	1.73±0.43	0.79±0.67
**NIP-4**	47.85±9.71	52.15±9.71	-	-
**NIP-4 loaded donepezil.HCl**	55.7±10.4	41.9±10.6	1.6±0.7	0.8±0.6
